# Elevated IFNA1 and suppressed IL12p40 associated with persistent hyperinflammation in COVID-19 pneumonia

**DOI:** 10.3389/fimmu.2023.1101808

**Published:** 2023-01-27

**Authors:** Kyeongseok Jeon, Yuri Kim, Shin Kwang Kang, Uni Park, Jayoun Kim, Nanhee Park, Jaemoon Koh, Man-Shik Shim, Minsoo Kim, Youn Ju Rhee, Hyeongseok Jeong, Siyoung Lee, Donghyun Park, Jinyoung Lim, Hyunsu Kim, Na-Young Ha, Hye-Yeong Jo, Sang Cheol Kim, Ju-Hee Lee, Jiwon Shon, Hoon Kim, Yoon Kyung Jeon, Youn-Soo Choi, Hye Young Kim, Won-Woo Lee, Murim Choi, Hyun-Young Park, Woong-Yang Park, Yeon-Sook Kim, Nam-Hyuk Cho

**Affiliations:** ^1^ Department of Microbiology and Immunology, Seoul National University College of Medicine, Seoul, Republic of Korea; ^2^ Department of Biomedical Sciences, Seoul National University College of Medicine, Seoul, Republic of Korea; ^3^ Department of Thoracic and Cardiovascular Surgery, Chungnam National University School of Medicine, Deajon, Republic of Korea; ^4^ Medical Research Collaborating Center, Seoul National University Hospital, Seoul, Republic of Korea; ^5^ Department of Pathology, Seoul National University College of Medicine, Seoul, Republic of Korea; ^6^ Department of Internal Medicine, Chungnam National University School of Medicine, Deajon, Republic of Korea; ^7^ Geninus Inc., Seoul, Republic of Korea; ^8^ Samsung Genome Institute, Samsung Medical Center, Seoul, Republic of Korea; ^9^ Chungnam National University Hospital, Biomedical Research Institute, Deajon, Republic of Korea; ^10^ Division of Healthcare and Artificial Intelligence, Department of Precision Medicine, Korea National Institute of Health, Korea Disease Control and Prevention Agency, Cheongju, Republic of Korea; ^11^ Department of Biohealth Regulatory Science, School of Pharmacy, Sungkyunkwan University, Suwon-si, Gyeonggi-do, Republic of Korea; ^12^ Biopharmaceutical Convergence Major, School of Pharmacy, Sungkyunkwan University, Suwon-si, Gyeonggi-do, Republic of Korea; ^13^ Department of Precision Medicine, Korea National Institute of Health, Korea Disease Control and Prevention Agency, Cheongju, Republic of Korea; ^14^ Institute of Endemic Diseases, Medical Research Center, Seoul National University, Seoul, Republic of Korea; ^15^ Seoul National University Bundang Hospital, Seongnam, Gyeonggi-do, Republic of Korea; ^16^ Wide River Institute of Immunology, Seoul National University, Hongcheon, Gangwon-do, Republic of Korea

**Keywords:** COVID-19, SARS-CoV-2, pneumonia, inflammation, IFNa, IL-12p40

## Abstract

**Introduction:**

Despite of massive endeavors to characterize inflammation in COVID-19 patients, the core network of inflammatory mediators responsible for severe pneumonia stillremain remains elusive.

**Methods:**

Here, we performed quantitative and kinetic analysis of 191 inflammatory factors in 955 plasma samples from 80 normal controls (sample n = 80) and 347 confirmed COVID-19 pneumonia patients (sample n = 875), including 8 deceased patients.

**Results:**

Differential expression analysis showed that 76% of plasmaproteins (145 factors) were upregulated in severe COVID-19 patients comparedwith moderate patients, confirming overt inflammatory responses in severe COVID-19 pneumonia patients. Global correlation analysis of the plasma factorsrevealed two core inflammatory modules, core I and II, comprising mainly myeloid cell and lymphoid cell compartments, respectively, with enhanced impact in a severity-dependent manner. We observed elevated IFNA1 and suppressed IL12p40, presenting a robust inverse correlation in severe patients, which was strongly associated with persistent hyperinflammation in 8.3% of moderate pneumonia patients and 59.4% of severe patients.

**Discussion:**

Aberrant persistence of pulmonary and systemic inflammation might be associated with long COVID-19 sequelae. Our comprehensive analysis of inflammatory mediators in plasmarevealed the complexity of pneumonic inflammation in COVID-19 patients anddefined critical modules responsible for severe pneumonic progression.

## Introduction

Coronavirus disease 2019 (COVID-19), caused by respiratory infection with severe acute respiratory syndrome coronavirus 2 (SARS-CoV-2), has spread worldwide with a disastrous impact on humankind. Currently, there have been more than 607 million infections globally, leading to over six million deaths due to acute respiratory distress syndrome (ARDS) as of September 2022 (https://covid19.who.int/). The pathogenesis of SARS-CoV-2-induced pneumonia is rather heterogeneous depending on clinical stage and may occur in two phases. First, the initial viral phase is characterized by viral replication resulting in direct virus-mediated tissue damage. Second, the extent of this damage response may sequentially determine the complex immunopathogenesis causing a local and systemic inflammatory response that can persist even after viral clearance (1, 2). Therefore, an optimal combination of antiviral and anti-inflammatory therapies may be required to prevent severe pneumonic progression and disease mortality in a stage- and severity-dependent manner (3). Further studies incorporating the impact of direct viral damage and sequential immunopathogenesis might be required to identify the best targets for early intervention and severity-specific treatment of COVID-19 since we have a limited understanding of key driving initiators of severe pulmonary inflammation and long COVID syndrome, also known as post-acute sequelae of SARS-CoV-2 infection.

In this study, we performed extensive quantitation of 191 proteins involved in various innate and adaptive immune responses and inflammation in plasma samples collected longitudinally from 347 confirmed COVID-19 pneumonia patients with well-defined clinical information and an additional 80 uninfected normal subjects. Plasma proteomics may reflect the integrated landscape of pulmonary and systemic inflammation in COVID-19 patients. Systemic analysis of kinetic changes and correlation according to disease stage and severity may also hold the promise of revealing causal relationships among the various inflammatory mediators. In addition, we confirmed their primary cellular sources based on single cell RNA (scRNA) sequencing data sets from lung autopsy and respiratory specimens. These results enabled us to define key inflammatory modules of molecular and cellular components involved in severe pneumonic progression, deduce their functional and causal relationship with stronger correlation power, and provide important insights into underlying mechanisms of driving effectors in severe COVID-19 patients in the context of relevant clinical outcomes. Therefore, our study may present key prognostic predictors required for biomarker development of effective therapeutics as well as advanced criteria for selecting patients for intensive care.

## Materials and methods

### Study design, patient information, and ethics statement

We enrolled 80 uninfected volunteers without respiratory disease and 347 SARS-CoV-2 PCR-positive patients admitted to Chungnam National University Hospital (Daejon, Republic of Korea), Seoul Medical Center (Seoul, Republic of Korea), and Samsung Medical Center (Seoul Republic of Korea). COVID-19 patients were categorized based on WHO severity definitions (https://covid19.who.int/) (4). General information on the baseline characteristics of the study participants included in this study are summarized in **Table 1**. Peripheral blood was collected in ethylenediaminetetraacetic acid (EDTA) tubes during hospitalization and centrifuged to collect plasma within 24 h after collection. Then, plasma samples were stored at -80°C before analysis. Lung autopsy samples were obtained from six deceased COVID-19 patients. Experiments conformed to the Declaration of Helsinki principles, and written informed consent was obtained from all donors or their legal guardians prior to the study. The clinical research was approved by the institutional review boards of Chungnam National University Hospital (IRB no.: CNUH 2020-12-002-008), Seoul Medical Center (IRB no.: SEOUL 2021-02-016), Samsung Medical Center (IRB no.: SMC-2021-03-160), Seoul National University Hospital (IRB no.: C-1509-103-705), and the Korea National Institute of Health (IRB no.: 2020-09-03-C-A).

**Table 1:** Demographics and baseline characteristics of COVID-19 patients.

Variables	Normal Control (N = 80)	COVID-19			p-value***
		Total (N = 347)	Moderate (N = 315)	Severe (N = 32)	
		WHO severity	Grade 4 (N = 280)	Grade 6-9 (N = 24)	
			Grade 5 (N = 35)	Grade 10 (N = 8)	
Sex, N (%)					0.791
male	40 (50.0%)	188 (54.0%)	171 (54.1%)	17 (53.1%)	
female	40 (50.0%)	159 (46.0%)	144 (45.9%)	15 (46.9%)	
Age, year					<0.0001
mean ± SD.	45.6 ± 16.9	53.5 ± 17.6	52.0 ± 17.3	68.1 ± 13.5	
range	21-78	19-92	19-92	36-91	
BMI, kg/m2					0.091
mean ± SD.	24.0 ± 3.6	24.2 ± 3.9	24.2 ± 3.9	23.4 ± 4.3	
range	17.0-31.0	12.4-39.4	12.4-39.4	15.9-31.2	
Comorbidity, N (%)					
Hypertension	18 (22.5%)	113 (32.7%)	94 (29.9%)	19 (59.4%)	0.0012
Diabetes	7 (8.8%)	78 (22.5%)	60 (19.1%)	18 (56.3%)	<0.0001
Cardiovascular ds.	2 (2.5%)	21 (6.1%)	16 (5.1%)	7 (21.9%)	0.1814
Respiratory ds.	0 (0.0%)	14 (4.0%)	10 (3.2%)	4 (12.5%)	0.0159
Kidney ds.	0 (0.0%)	13 (3.8%)	11 (3.5%)	2 (6.3%)	0.1474
Other chronic ds.*	1 (1.3%)	31 (9.0%)	26 (8.3%)	5 (15.6%)	<0.0001
Treatment, N (%)					
Antibiotics		56 (16.2%)	44 (14.0%)	12 (37.5%)	
Antiviral drugs		44 (12.7%)	23 (7.3%)	19 (59.4%)	
Corticosteroids		73 (21.1%)	43 (13.7%)	30 (93.8%)	
Other therapies**		101 (29.2%)	93 (29.6%)	8 (25%)	
Time from onset to O2 therapy, Days					
mean ± SD.		7.1 ± 4.0	7.4 ± 3.6	6.7 ± 4.4	
range		1-18	1-18	1-18	

*neoplasia, chronic liver ds., or dementia.

**Immune plasma, monoclonal antibodies, anticoagulant, or Pyramax. ***One-way ANOVA was used to estimate p-values for significant difference in demographic features among normal control, moderate, and severe groups.

### Multiplex immunoassay of plasma proteins

To identify the differentially regulated plasma factors depending on COVID-19 disease severity, 350 plasma factors (**Supplementary Table S1**) from 20 plasma samples (10 from 3 moderate patients and 10 from 5 severe patients) were screened by quantitative immunoassays using a total of 21 multiplex panels according to the manufacturers’ instructions *via* a multiplex assay service (Koma Biotech., Seoul, Republic of Korea). Four types of commercially available kits were used for measurement (**Supplementary Table S1**). The MILLIPLEX MAP Human Complement Magnetic Bead Panel 2 (Millipore, Burlington, MA, USA) included C1q, C3, C3b/iC3b, C4, complement factor B, complement factor H, and properdin. The MILLIPLEX MAP Human Sepsis Magnetic Bead Panel 3 (Millipore) included elastase 2, lactoferrin, NGAL, resistin, and thrombospondin-1. Magnetic Luminex Performance Assay multiplex kits (R&D Systems, Inc. Minneapolis, MN, USA) were used for TGF-β1-3. Magnetic Luminex Screening Assay multiplex kits (R&D systems, Inc.) included all the other factors measured in this study. Assay plates were read with a Luminex 100/200TM analyzer (ThermoFisher, Waltham, MA, USA). For quantification for each factor, the supplied standard proteins were used, and a standard curve was drawn by the best fit algorithm using MasterPlex QT 2010 software (MiraiBio, Hitachi, CA, USA). We used detection limit values of non-detected factors below the detection range (**Supplementary Table S2**). Based on the screening results, 191 plasma factors were selected for further studies (**Supplementary Tables S3**).

### Lung tissue preparation, H&E staining, and scRNA sequencing

Lung autopsy samples obtained from deceased patients were immediately fixed in 10% formalin or immersed in RNAlater solution (ThermoFisher) for paraffin embedding or scRNA sequencing, respectively. Paraffin-embedded lung tissue samples were prepared as previously reported (5). Briefly, the tissues that were fixed overnight were dehydrated and defatted by immersing in ethanol and xylene sequentially and treated with melted paraffin (58-60°C). Paraffin-embedded tissues were cut at a thickness of 4 μm and stained with hematoxylin and eosin (H&E). Lung pathology was evaluated and analyzed by two experienced pathologists under a light microscope (BX-53, Olympus, Tokyo, Japan). For scRNA sequencing, lung tissues were dissociated into single cells by chopping with a blade and were incubated in RPMI1640 containing 1 mg/ml Collagenase IV (ThermoFisher) and 0.1 mg/ml DNase I (Worthington, Columbus, OH, USA) at 37°C for 30 min. Lung single cells were filtered by nylon mesh and 70 μm cell strainers (Falcon) and centrifuged at 1,000 x *g* for 5 min. After RBC lysis, cell counts and viability were measured with a Countess 3 Automated Cell Counter (ThermoFisher), and 20,000 live cells were used to generate gel beads-in-emulsion (GEMs) by using the Chromium Single Cell 5’ Library and Gel Bead Kits v.1 and a Chromium Controller (10x Genomics) according to the manufacturer’s instructions, as we previously reported (6). After GEM-RT incubation and cDNA amplification, the cDNA quality and concentration were analyzed and calculated using an Agilent Bioanalyzer (Agilent Technologies, Santa Clara, CA, USA), and scRNA sequencing was performed using the NextSeq 550 platform (Illumina, San Diego, CA, USA).

### Bioinformatics of scRNA-seq and statistical analysis

The raw sequencing data for scRNA-seq were processed with CellRanger (version 3.1.0) (7). Reads were aligned to the combined genome of human (GRCh38, Ensembl) and SARS-CoV-2 (ASM985889v3, NCBI). The feature-barcode matrices were generated using the CellRanger count. The cells of the feature-barcode matrices were filtered by the numbers of expressed genes and the mitochondrial-to-total gene count ratio. The filtered feature-barcode matrices were used to create Seurat (version 4.1.1) objects (8). The Seurat objects were normalized using the SCTransform algorithm. To align the cells originating from different samples, 3,000 highly variable genes from each sample were selected. Anchors representing a similar biological state across samples based on the overlap in their nearest neighbors were sought, and samples were aligned based on the top 20 canonical correlation vectors. The aligned samples were scaled, and principal component analysis (PCA) was conducted. The cells were clustered by unsupervised clustering (0.2 resolution) and visualized by UMAP. To identify marker genes, upregulated genes in each cluster relative to the other clusters were selected on the basis of the Wilcoxon rank sum test implemented in Seurat FindAllMarkers function with >0.25 log fold change compared with other clusters and a Bonferroni-adjusted *P* < 0.05. By manual inspection, the 17 different clusters were assigned to 13 cell types.

Unsupervised clustering of samples, patients, or plasma factors was performed using the *k*-means algorithm. The optimum number of clusters was determined by using silhouette coefficient analysis in NBClust and factoextra packages (R package Version 1.0.7.). Before data visualization, each feature was scaled and centered as a z score using the scale function in R software. Multiple group comparisons were performed using the two-tailed Mann−Whitney test or Kruskal−Wallis test. Spearman’s correlation test was performed using the corrplot package in R software. For visualization, heatmaps and dot plots were generated using the ComplexHeatmap (9) and ggplot2 packages, respectively. Correlation plots were generated with the corrplot package by only showing correlations with *p* < 0.05 and ordered by hierarchical clustering. Core I and core II in the global correlation network are indicated based on hierarchical clustering results in the correlation plot.

### Gene ontology and pathway enrichment analysis

Plasma factors were considered to be expressed differentially if there were significant differences (*p* < 0.05) in comparison among the NC group, moderate group (grades 4 and 5), and severe group (grades 6~10). These differentially expressed proteins were subjected to gene set enrichment analysis to assess the biological function related to COVID-19 severity. Enrichment analysis of GO biological pathways and hallmark gene sets was performed using the clusterProfiler (10) and enrichR (11) packages of R statistical software (R core team, 2020), respectively. Enriched terms were visualized using ggplot2 (12).

### Linear mixed model analyses

Demographic and baseline characteristics were expressed as the mean with standard deviation and range for continuous variables and frequencies with percentages for categorical variables. Differences among group severities in plasma factors were assessed with independent samples *t* test or Wilcoxon rank sum test according to their normality. In addition, if the results of group comparison were statistically significant, pairwise multiple comparison with Bonferroni correction was applied. We performed hierarchical clustering with all plasma factors to distinguish biologically distinct subgroups with a distance-based algorithm. A linear mixed model was used to investigate the periodically measured plasma factor changes over time, adjusting for age and sex. Statistical analysis was conducted using SAS 9.4 software (SAS system for Windows, version 9.4; SAS Institute, Cary, NC, USA) and the R package (version 4.2.1, R Core Team, 2020; R: A language and environment for statistical computing. R Foundation for Statistical Computing, Vienna, Austria. URL https://www.R-project.org/).

### Quantitation of viral loads

Real-time reverse transcription-polymerase chain reaction (RT−PCR) assays for the detection of SARS-CoV-2 were performed according to the manufacturer’s instructions (Kogenebiotech, Seoul, Republic of Korea) (5). Total RNA was obtained from nasopharyngeal and throat swab (upper respiratory tract) and sputum (lower respiratory tract) samples. Primer sets targeting the E and RdRP genes of SARS-CoV-2 were used with a cutoff cycle threshold (Ct) value higher than 38 cycles.

## Results

### Patient characteristics

**Figure 1:**
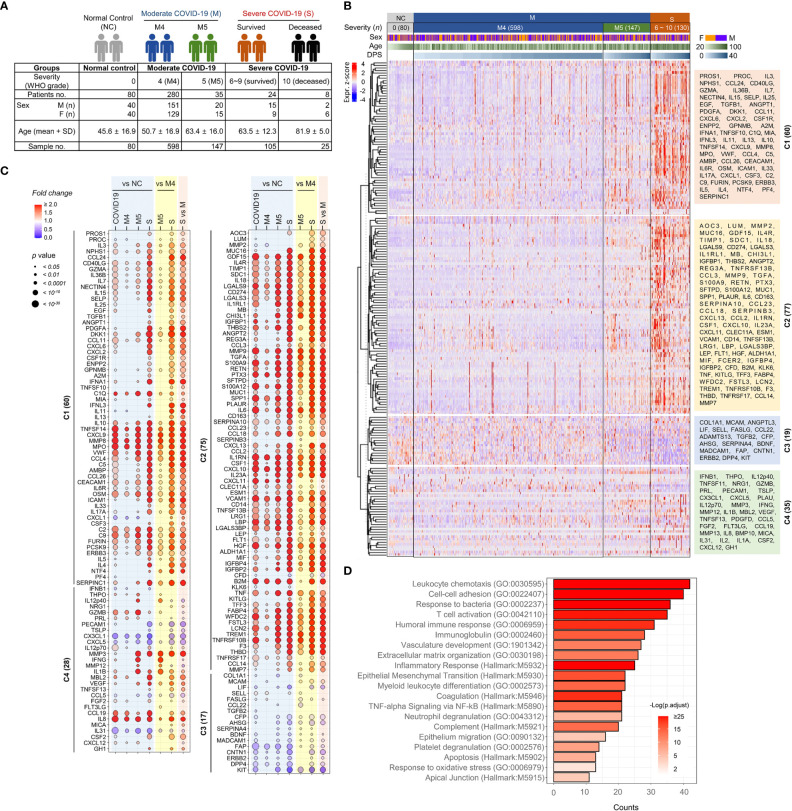
Overt inflammatory responses associated with severe COVID-19 pneumonia. **(A)** Overview of the study cohort. **(B)** Unsupervised hierarchical clustering of 191 plasma factors measured by multiplexed immunoassay presented four separated clusters, C1-C4. The plasma samples are arranged according to COVID-19 severity (M: moderate pneumonia, S: severe pneumonia) and the collection days post-symptom onset (DPS). Eighty plasma samples collected from uninfected volunteers (NCs) are also included. The heatmap shows z scores and clustering determined using correlation and average linkage. **(C)** Bubble plots show the relative fold change in the average level of each plasma factor in comparison with indicated patient groups. The statistical significance in difference among the subject groups was calculated with the Mann–Whitney test or Kruskal−Wallis test. **(D)** Gene Ontology (GO) and hallmark gene set-based enrichment analyses of differentially regulated plasma proteins among NCs and COVID-19 patients present representative biological processes and the number of counts with statistical significance.

The baseline characteristics of the confirmed COVID-19 patients included in this study are summarized in **Table 1** and **Figure 1A**. The uninfected normal control (NC) group included 80 sex- and age-matched individuals who provided 80 plasma specimens. A total of 347 hospitalized patients with confirmed COVID-19 pneumonia participated in our cohort and were classified based on WHO clinical grading (grade 4-10: 4, moderate disease without oxygen therapy; 5, moderate COVID-19 with oxygen therapy by mask or nasal prongs; 6, severe disease treated with noninvasive ventilation or high flow oxygen; 7, severe disease with intubation and mechanical ventilation [pO_2_/FiO_2_ ≥ 150 or SpO_2_/FiO_2_ ≥ 200]; 8, severe disease with mechanical ventilation [pO_2_/FiO_2_ < 150 or SpO_2_/FiO_2_ < 200] or vasopressors; 9, severe COVID-19 treated with mechanical ventilation [pO_2_/FiO_2_ < 150] and vasopressors, dialysis, or extracorporeal membrane oxygenation; and 10, deceased patients) (https://covid19.who.int/) (4). The moderate group with grade 4 (M4) or 5 (M5) COVID-19 included 280 and 35 patients, respectively. The severe COVID-19 group with grades 6 to 10 (S6 ~ S10) included 32 patients, with 8 deceased patients due to fatal ARDS. The NC, moderate, and severe COVID-19 groups had similar proportions of male and female patients, but the age distribution in the severe group (mean ± S.D.: 68.1 ± 13.5) was older than those in the NC (45.6 ± 16.9) and moderate groups (52.0 ± 17.3). The severe group presented a significantly higher proportion of comorbidities, such as hypertension and diabetes, than the NC and moderate groups (**Table 1**). All patients were recruited before July 2021 and were not immunized with the COVID-19 vaccine before infection.

First, we investigated the potential association of disease severity with viral loads in respiratory secretions (13). The overall viral loads in the upper (nasopharyngeal swab specimens) and lower (sputum specimens) respiratory tract samples were not significantly different between the moderate and severe groups (**Supplementary Figure S1A-C**). When we assessed the potential effect of age on viral dynamics, overall viral loads tended to be similar among the patient groups regardless of age (old: ≥60, young: <60) and severity (**Supplementary Figure S1C**), even though older patients in the M4 group showed significantly higher viral loads than older patients in the severe group, especially based on lower respiratory tract specimens.

### Overt inflammatory responses associated with severe COVID-19 pneumonia

To screen plasma factors differentially regulated according to the disease severity of COVID-19, we performed quantitative immunoassays using 21 multiplex panels detecting 350 plasma factors that are functionally associated with various types of inflammation and immune responses (**Supplementary Table S1**). In the pilot test, 20 plasma samples (10 from 3 moderate patients and 10 from 5 severe patients) collected after symptom onset were analyzed (**Supplementary Table S2**), and we selected 191 plasma factors (**Supplementary Table S3**), namely, 60 factors showing significant differences between the samples from the moderate and severe groups in the initial screening and 131 plasma proteins potentially associated with pulmonary and systemic inflammation reported in previous studies (14–19). Plasma samples were longitudinally collected from 347 COVID-19 patients 1~6 times at 3~7-day intervals. Quantitative analysis of the 191 selected factors in 955 plasma specimens from 80 NCs (*n*=80 plasma samples) and 347 COVID-19 patients (*n*=875 plasma samples) was performed, and the results are summarized in **Figure 1B**. Unbiased hierarchical clustering of the Z-score trajectories of all the plasma factors demonstrated four clearly separated major clusters (C1 ~ C4). C1 and C2 were generally elevated in severe patients when compared to NCs and moderate patients, whereas the C3 group tended to be decreased in severe patients (**Figure 1C**). The C1 factors showed more persistent responses, but the C2 factors tended to be decreased gradually at later stages. The C4 group factors presented heterogeneous responses with fluctuations depending on disease severity and course. A comparison of the overall mean values of the plasma factors among the NC and patient groups revealed that 180 factors were significantly and differentially regulated (**Figure 1C**). A comparison of the mean values between the NC and COVID-19 groups revealed that 153 plasma factors were significantly and differentially regulated (128 factors upregulated and 25 factors downregulated in COVID-19 patients). When we performed linear mixed model analysis to assess significant differences among the severity groups after adjusting for the age and sex of the patients to further confirm the specific association of the plasma factors with disease severity (**Supplementary Table S4**), 167 factors showed significant differences between the NC and COVID-19 groups and their time-dependent trajectories, even after adjusting for age and sex. Differential expression analysis between the moderate and severe COVID-19 groups indicated that 169 factors were significantly upregulated (145 factors) or downregulated (24 factors) in plasma from severe COVID-19 patients compared to moderate pneumonia patients (**Figure 1C**). Even though most of the differentially expressed plasma factors were gradually increased or decreased depending on disease severity, it is noteworthy that several factors, including IL12p40, GZMB, IFNG, MMP12, and IL1B, were significantly upregulated in the M5 group compared with the NC, M4, or severe COVID-19 groups (**Figure 1C**).

The 180 differentially regulated plasma factors in the NC and COVID-19 groups were subjected to Gene Ontology enrichment and hallmark gene set enrichment analyses (20, 21). Even though our quantitative assay was based on selected panels mainly related to immune responses and inflammation, relative enrichment of specific pathways was observed (**Figure 1D**). These included pathways primarily involved in leukocyte chemotaxis, cell−cell adhesion, response to bacterial molecules, T-cell activation, humoral immune response, vascular development, extracellular matrix organization, and epithelial-mesenchymal transition.

**Figure 2:**
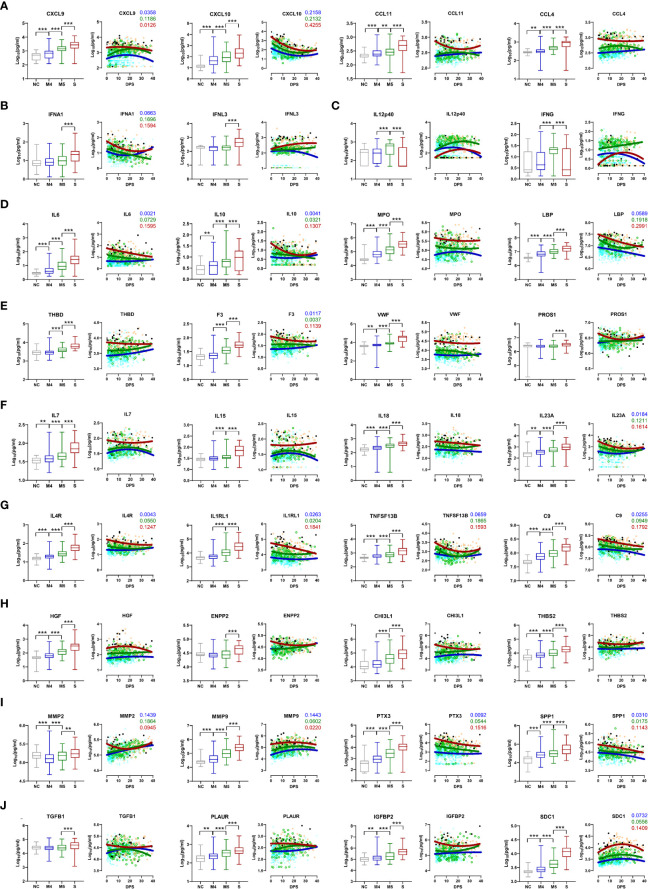
Kinetic changes in representative plasma factors differentially regulated among NCs and COVID-19 patients. Kinetic responses of representative inflammatory factors involved in leukocyte chemotaxis **(A)**, type I and III interferon response **(B)**, Th1 cytokines **(C)**, general inflammation **(D)**, endothelial activation and coagulation **(E)**, T-cell homeostasis and activation **(F)**, humoral response **(G)**, vascular development **(H)**, extracellular matrix organization **(I)**, and epithelial-mesenchymal transition **(J)**. The overall protein level of an indicated factor in plasma is compared among the NC and patient groups with the Kruskal−Wallis test (left panels) and their kinetic responses according to days post-symptom onset (DPS) (right panels). Solid lines indicate nonlinear regression. R squared values are colored accordingly if the value of any regression is above 0.1. Gray: NC, blue: M4 group, green: M5 group, red: severe group, and black dots for fatal cases. **p < 0.01, ***p < 0.001.

The kinetic responses of 170 plasma proteins showing significant differences among the NC, M4, M5, and severe groups are presented in **Supplementary Figure S2**. Kinetic changes in representative factors involved in the enriched functional pathways showed significant differences among the patient groups, as summarized in **Figure 2**. For example, a type I interferon, IFNA1, presenting a significantly higher response in severe COVID-19 patients, surged during the early phase of symptom onset and gradually declined, whereas IFNL3, a type III interferon, which was also significantly elevated in the severe group compared with the moderate group, gradually increased in severe patients during disease progression (**Figure 2B**). Other plasma factors involved in inflammation (IL6, IL10, IL13, MPO, and LBP), endothelial activation and coagulation (THBD, F3, VWF, PROS1, and MMP8), T-cell homeostasis and activation (IL7, IL15, IL18, IL4, and IL23A), and humoral response (IL4R, IL1RL1, IL33, TNFSF13B, and C9) were also significantly upregulated with various kinetic responses (**Figures 2D–G**). These overt inflammatory responses associated with severe disease progression were concomitantly presented with elevated tissue damage responses, such as vascular development (HGF, ENPP2, CHI3L1, THBS2, and ANGPT2), extracellular matrix organization (MMP2, MMP3, MMP9, PTX3, and SPP1), and epithelial mesenchymal transition (TGFB1, PLAUR, IGFBP2, IGFBP4, and SDC1) (**Figures 2H–J**), suggesting the complexity of dysregulated systemic inflammation potentially initiated from severe pulmonary insults by SARS-CoV-2 infection (5, 22, 23).

### Cellular sources of plasma factors in the inflamed lungs of COVID-19 patients

Histopathologic examination of the lungs of patients who died due to COVID-19 revealed variable phases of diffuse alveolar damage from the acute exudative phase to the proliferative and fibrotic phases (**Supplementary Figure S3**). The pathologic findings from the lungs included diffuse interstitial thickening, fibrosis, granulation tissue formation and lymphoplasma cell infiltration with reactive type II pneumocyte hyperplasia and hyaline membrane formation in the alveolar wall. Occasional foci of macrophages, neutrophil infiltration with seromucinous fluid or hemorrhage in alveolar spaces were also observed. Focal pulmonary microthrombi or large vessel thrombi were identified in some patients.

SARS-CoV-2 RNA was barely detected in our autopsy samples by scRNA sequencing analysis, and 13 cell types were identified and manually annotated (**Supplementary Figure S4, S5A**). Macrophages were the predominant inflammatory cells, ranging from 47.9% to 78.7% of lung-infiltrating leukocytes. T cells were the second most dominant cell type, comprising 4.2% to 28.1%, and neutrophils accounted for 7.4~14.5% of the pulmonary leukocytes. NK and NKT cells represented approximately 5.2% and 1.9% of the pulmonary leukocytes, respectively, and B cells accounted for ~ 1.1% (**Supplementary Figure S5B**). We also observed that 0.7~3.0% of the leukocytes were mast cells in the lung autopsy samples from the patients who died. Some notable differences between the NC and fatal COVID-19 lungs were a relative decrease in NK cells in the patients (0.5–9.7%, mean=5.7%) *vs*. controls (mean: 16.1%) as well as an increase in macrophages (47.9–78.7%, mean=59.4%) and NKT cells (0.2–4.5%, mean=1.9%) in COVID-19 patients *vs*. controls (mean=43.7% and 0.4% for macrophages and NKT cells, respectively), even though the differences were not statistically significant. To further examine the respiratory leukocyte population in COVID-19 patients, we utilized two public scRNA data sets based on analyses of respiratory samples (nasopharyngeal and bronchoalveolar lavage fluid samples) (**Supplementary Figure S6A**) (24, 25). These included 9 NC sets and 36 COVID-19 samples from 28 patients (11 moderate and 17 severe patients, including 4 fatal cases). The relative proportions of specific leukocyte populations in the combined data set from COVID-19 patients revealed remarkable reductions in NK (moderate: 12.8%, severe: 2.6%) and T (moderate: 11.3%, severe: 5.1%) cell populations in the severe COVID-19 group compared to the moderate COVID-19 group (**Supplementary Figure S6B**). In contrast, neutrophils were increased in severe patients (30.1%) compared with moderate patients (19.5%). In addition, NKT cells (moderate: 5.5%, severe: 6.9%), B cells (moderate: 2.6%, severe: 3.7%), and macrophages (moderate: 48.2%, severe: 51.6%) were slightly increased in the severe group compared with the moderate group.

When we examined transcriptional expression of the plasma factors in the scRNA data sets to identify the cellular sources of the plasma proteins, we detected 180 transcripts among 191 factors in the scRNA data sets from our lung autopsy samples or previously deposited respiratory samples (24, 25). RNA transcripts for 124 plasma factors were detected in more than 10% of a specific cellular type and are summarized in **Supplementary Figure S5C, S6C**.

### Global correlation map of 191 plasma proteins in COVID-19 patients

**Figure 3:**
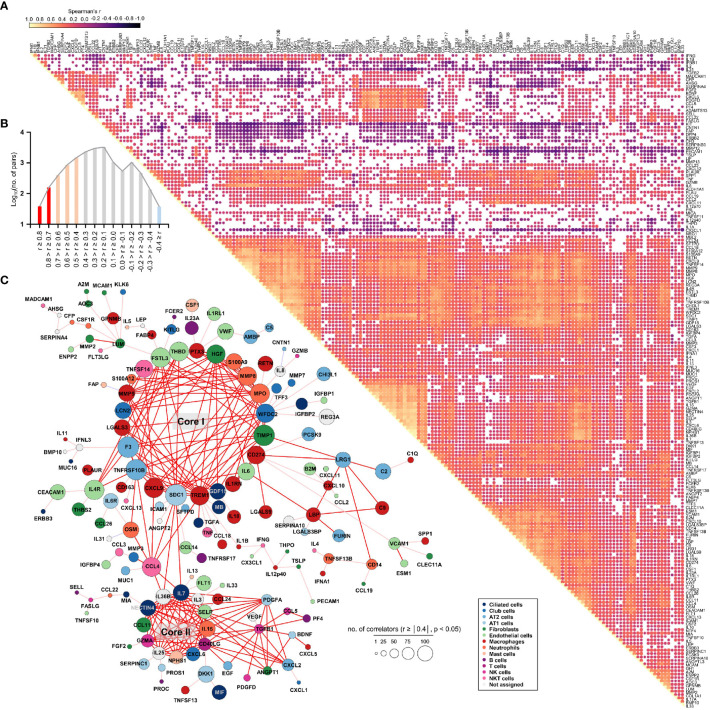
Global correlation map of 191 plasma proteins from COVID-19 pneumonia patients. **(A)**, Correlation matrix across all time points of 191 plasma factors from COVID-19 patients. Only significant correlations (*p* < 0.05) are represented as dots. Spearman’s correlation coefficients from comparisons of protein measurements within the same specimen are visualized by color intensity. **(B)** Distribution of the number of significant correlation pairs (*p* < 0.05). Red (robust positive correlation): Spearman’s *r* ≥ 0.7, orange (moderate positive correlation): 0.7 > Spearman’s *r* ≥ 0.4, and sky blue (moderate negative correlation): -0.7 < Spearman’s *r* ≤ -0.4. There was no robust negative correlation pair (Spearman’s *r* ≤ -0.7). **(C)** Global correlation map of 159 proteins based on the correlation coefficients and the number of significant correlators of each plasma factor displaying an absolute Spearman’s *r* ≥ 0.4. The circle size is proportionally adjusted depending on the number of significant correlators, and the color code of each component is determined based on primary cellular source (see **Supplementary Figure S5C, S6C**). All the correlation pairs with Spearman’s *r* ≥ 0.7 are linked by a red line. If the absolute r value of maximum correlation was more than 0.4 but less than 0.7, only the best correlator is linked by either a pink line (positive corr.) or a sky blue line (negative corr.).

To assess the potential associations of all 191 quantified proteins with each other in COVID-19 patient plasma, we generated a global correlation map (**Figure 3**). This consists of the pairwise correlation of 191 plasma factors in 875 patient samples (36,481 correlation coefficients) that were subjected to unsupervised hierarchical clustering (**Figure 3A**). This approach revealed 14,801 significantly (*p* < 0.05) correlated pairs showing either positive (12,584 pairs) or negative (2,217 pairs) correlations (**Figure 3B**). Among the significantly correlated pairs, 195 pairs presented robust positive correlation (Spearman’s *r* ≥ 0.7), and 2,745 pairs displayed moderate positive correlation (0.7 > Spearman’s *r* ≥ 0.4), whereas only 37 pairs showed moderate negative correlation (-0.7 < Spearman’s *r* ≤ -0.4). Based on the correlation coefficients and the number of significant correlators of each plasma factor displaying absolute Spearman’s *r* ≥ 0.4, we generated a global correlation map including 159 proteins (**Figure 3C**). We also annotated the primary cellular sources of each factor, as shown in **Supplementary Figure S5C, S6C**. The global correlation map presenting robust correlation (Spearman’s *r* ≥ 0.7 in red lines) highlighted two core modules, cores I and II. The core I module comprised 23 plasma factors mainly derived from macrophages (PTX3, MMP8, MPO, TIMP1, CD274, IL6, IL1RN, TREM1, CXCL9, LGALS3, and MMP9), neutrophils (PTX3, S100A9, MMP8, MPO, TIMP1, CD274, IL1RN, TREM1, MMP9, and S100A12), epithelial cells (WFDC2, GDF15, SDC1, TNFRSF10B, F3, LGALS3, LCN2, and FSTL3), and endothelial cells (IL6, FSTL3, and THBD) (**Figure 3C**). The core II module included 12 proteins primarily expressed in epithelial cells (IL7, IL36B, NECTIN4, and CXCL6), NK(T) and T cells (GZMA, CD40LG, and IL3), and fibroblasts (CCL11) (**Figure 3C**). The core I and II components displayed robust and multiple correlations with each other and significant correlations with approximately 170 factors on average, suggesting a strong and wide functional relationship. In addition, several core I factors, such as TIMP1, CD274, and IL6, were strongly correlated with LRG1 and LBP derived from epithelial cells and macrophages, respectively, as well as complement factors C2 and C9. Components of core II also strongly correlated with PDGFA, CXCL2, and TGFB1, which were primarily expressed in alveolar epithelial cells and T cells, respectively. Notably, CCL4 derived from NKT and T cells showed a strong positive correlation with several components of the core I and core II networks, suggesting a connective role between both inflammatory networks (**Figure 3C**).

**Figure 4:**
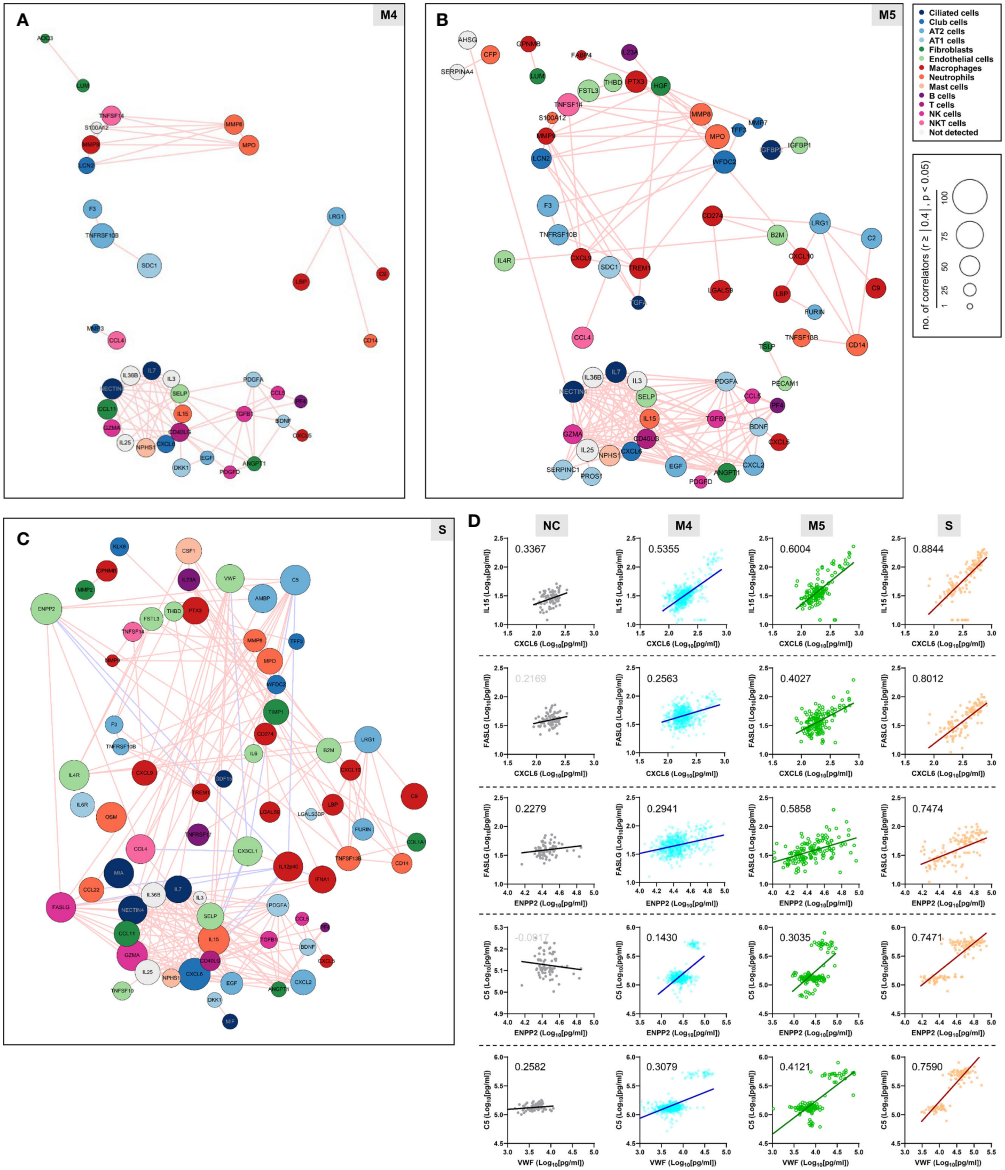
Enhanced correlation in quantity and quality among the plasma factors according to COVID-19 severity. Correlation maps of plasma proteins displaying robust correlation power (absolute Spearman’s *r* ≥ 0.7) in plasma samples from moderate **(A)** M4 and **(B)** M5 and severe patients **(C)** S are presented. The circle size is proportionally adjusted depending on the number of significant correlators (absolute Spearman’s *r* ≥ 0.4), and the color code of each component is determined based on primary cellular source. All the correlated pairs with a Spearman’s r ≥ 0.7 are linked by either pink (positive corr.) or sky blue lines (negative corr.). **(D)** Representative correlation plots of the indicated plasma factors which show enhanced correlation power in quantity and quality according to COVID-19 severity. Spearman’s *r* value for each plot is presented.

**Figure 5:**
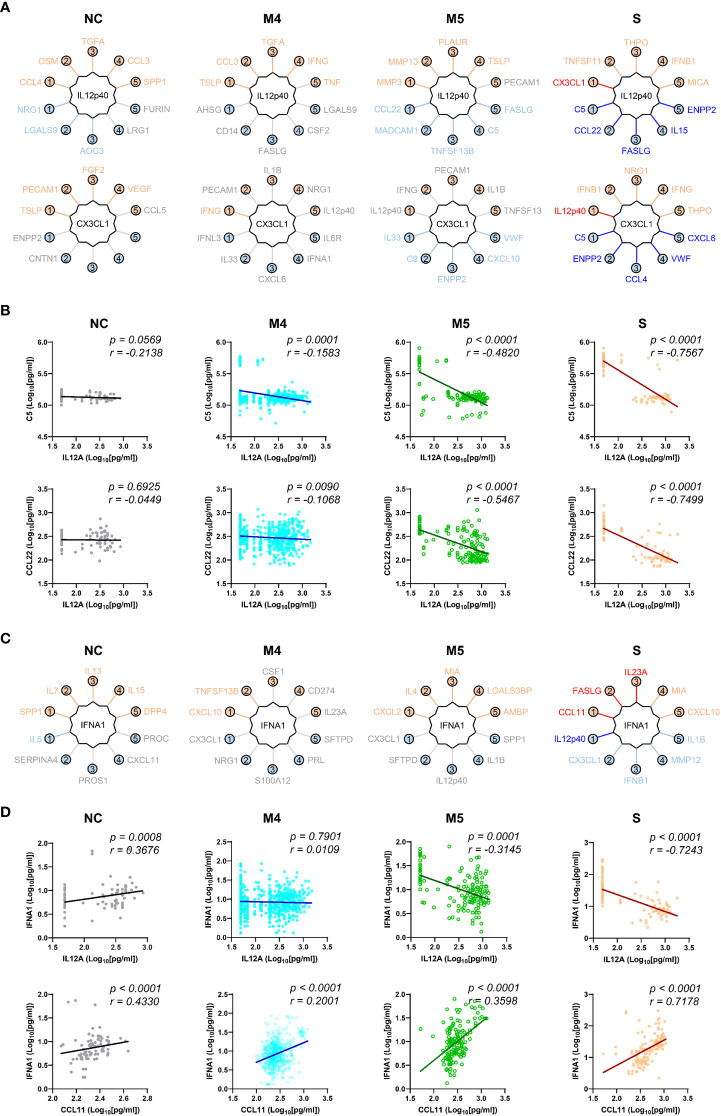
Robust inverse correlation of IL12p40 and CX3CL1 with various inflammatory mediators and IFNA1 in severe COVID-19 patients. **(A)** Top 5 positive and negative correlators of IL12p40 and CX3CL1 in NC and COVID-19 patients with various disease severities (M4 and M5: moderate pneumonia, S: severe pneumonia). Upper factors are the best 5 positive correlators and lower mediators are the best 5 negative correlators of IL12p40 and CX3CL1. The color of each factor is annotated according to the value of their correlation coefficient; red and blue: robust correlation with absolute Spearman’s *r* ≥ 0.7, orange and sky blue: moderate correlation with 0.7 > absolute Spearman’s *r* ≥ 0.4, and gray: no significant correlation (*p* > 0.05). **(B)** Representative correlation plots of IL12p40 with C5 (upper panels) and CCL22 (lower panels). Spearman’s *r* and *p* value for each plot are presented. **(C)** Top 5 positive and negative correlators of IFNA1 in NC and COVID-19 patients with various disease severities (M4 and M5: moderate pneumonia, S: severe pneumonia). Color code of each factor is applied as in **(A, D)**. Representative correlation plots of IFNA1 with IL12p40 (upper panels) and CCL11 (lower panels). Spearman’s *r* and *p* value for each plot are presented.

We next examined changes in patterns of the correlation network in each severity group to assess the impact of plasma factors according to COVID-19 severity (**Figure 4** and **Supplementary Figure S7**). The total number of significant correlation pairs with absolute Spearman’s *r* ≥ 0.4 gradually increased in a severity-dependent manner, and the severe group presented 3,121 correlated pairs, whereas the M4 group and M5 group had 1,576 and 2,086 significant correlated pairs, respectively, suggesting stronger and more diverse functional associations of the plasma factors during more severe disease progression (**Supplementary Figure S7B**). The global correlation map of the M4 and M5 groups (moderate pneumonia) with the robust correlated pairs (absolute Spearman’s *r* ≥ 0.7) included 39 and 66 proteins, respectively (**Figures 4A, B**). The severe group presented 72 factors with robust correlations, and the number of significantly (absolute Spearman’s *r* ≥ 0.4) correlated pairs of robust correlators (*n*=1,975) was drastically increased when compared with those of the M4 (*n*=710) and M5 (*n*=1,191) groups (**Figure 4C** and **Supplementary Figure S7B**). We observed that the number of robust correlators in the core I module in the M5 (*n*=17) and severe (*n*=16) groups was higher than that in the M4 group (*n*=9). In contrast, the robust correlators in the core II module seemed to be rather conserved regardless of disease severity, but the number of significantly correlated pairs (absolute Spearman’s *r* ≥ 0.4) was increased, especially those of CXCL6, IL15, GZMA, and NECTIN4. These results suggest that pneumonic progression requiring oxygen supply (M5 and severe groups) might be facilitated by more diverse inflammatory mediators in the core I module, whereas critical pneumonic commitment requiring intensive respiratory care (severe group) may be associated with enhanced impact on other mediators by the members of the core II module. Additionally, several factors, such as ENPP2, C5, FASLG, VWF, IFNA1, and CSF1, newly appeared as robust positive correlators strongly associated with more diverse plasma factors in the severe group than in the moderate group (**Figure 4C**), also indicating their enhanced impact on other plasma factors during severe pneumonic progression in COVID-19 patients. Consistently, we observed a gradual increase in correlation power among the top correlators, such as IL-15, CXCL6, FASLG, ENPP2, C5, and VWF, in the severe group as the disease severity was aggravated (**Figure 4D**). Furthermore, we noticed that IL12p40 and CX3CL1 displayed strong positive correlations with each other but showed robust negative correlations with other inflammatory mediators, such as C5, ENPP2, CCL22, FASLG, CCL4, VWF, IL15, and CXCL6, in the severe patient group (**Figure 4C**, **5**, and **Supplementary Figure S8**). The degree of negative correlation of IL12p40 and CX3CL1 with inflammatory factors gradually increased depending on COVID-19 severity (**Figure 5A, B**), suggesting that suppression of IL12p40 and CX3CL1 might be associated with enhanced inflammatory responses driving critical pneumonic progression in patients. In addition, IL12p40 was the top negative correlate of IFNA1 in the severe group, and their inverse correlation was gradually enhanced according to COVID-19 severity (**Figures 5C, D**). IFNA1 also presented a robust positive correlation with CCL11, FASLG, and IL23A in severe patients, and their correlation power was gradually enhanced as COVID-19 severity increased.

### Association of elevated IFNA1 and suppressed IL12p40 with hyperinflammation in COVID-19 pneumonia

**Figure 6:**
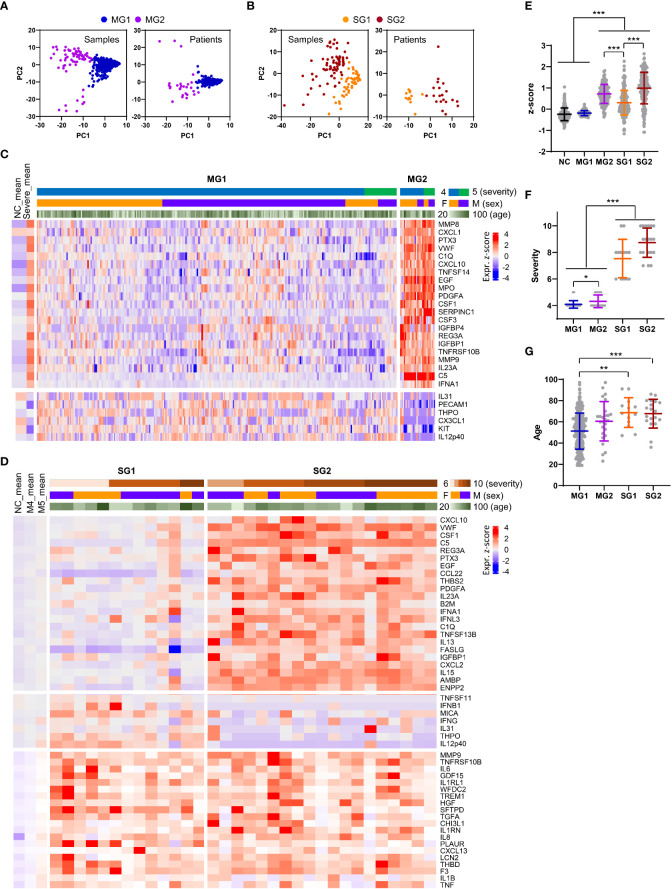
Heterogeneity of inflammatory responses in COVID-19 pneumonia patients. **(A, B)** PCA of plasma proteins in moderate (MG1 and MG2, **(A)** and severe (SG1 and SG2, 9B) COVID-19 patients. Each dot represents 1 plasma sample or the mean value of a single patient. **(C)** Z-score heatmap with unsupervised clustering of the mean concentrations of plasma factors defines the MG1 and MG2 groups of moderate COVID-19 patients. Representative plasma proteins differentially expressed between the MG1 and MG2 groups were selected and presented. The average z scores of the NCs (NC_mean) and severe patients (severe_mean) are shown in the left panel. **(D)** Z-score heatmap with unsupervised clustering of the mean concentration of plasma factors defines the SG1 and SG2 groups of severe COVID-19 patients. Representative plasma proteins differentially expressed between the SG1 and SG2 groups or significantly elevated in both severe groups when compared to the moderate groups were selected and presented. The average z scores of the NCs (NC_mean) and moderate patients (M4_mean and M5_mean) are shown in the left panel. **(E)** Distribution of the mean z scores of 191 factors in the NC group, moderate groups (MG1 and MG2), and severe groups (SG1 and SG2). **(F)** Distribution of disease severity in the moderate groups (MG1 and MG2) and severe groups (SG1 and SG2). **(G)** Age distribution in the moderate groups (MG1 and MG2) and severe groups (SG1 and SG2). Statistical significance among the patient groups was calculated with the Kruskal−Wallis test (**p < 0.01, ***p < 0.001).

When we examined the plasma factors in the plasma samples or patient data sets with average values from the moderate patients by principal component analysis (PCA) and unbiased clustering, there were two separate groups, MG1 (*n*=289, 91.7%) and MG2 (*n*=26, 8.3%) (**Figures 6A, C**, and **Supplementary Figure S9**). Interestingly, MG2 patient samples presented consistent elevation of a group of plasma factors regardless of plasma collection time (**Supplementary Figure S9A**). Even though this does not clearly differentiate the disease severity, MG2 patients tended to present significantly higher levels of inflammatory mediators than MG1 patients (**Figure 6E**) and associated with more severe COVID-19 than MG1 patients (**Figure 6F**). Comparison of the mean values between MG1 and MG2 patients revealed that 155 plasma factors were significantly and differentially regulated (145 factors upregulated and 10 factors downregulated in MG2 patients) (**Supplementary Table S5**). Among them, MMP8 (7.8-fold), CXCL1 (7.0-fold), PTX3 (6.4-fold), VWF (5.0-fold), C1Q (4.9-fold), CXCL10 (4.8-fold), TNFSF14 (4.7-fold), EGF (4.6-fold), MPO (4.6-fold), PDGFA (4.6-fold), CSF1 (4.6-fold), SERPINC1 (4.6-fold), and CSF3 (4.3-fold) in MG2 patients compared to MG1 patients were upregulated by more than four times on average (**Figure 6C**). In addition, IFNA1 was significantly elevated in MG2 patients by 2.8-fold compared with MG1 patients. In contrast, IL12p40 (0.3-fold), KIT (0.3-fold), and CX3CL1 (0.3-fold) were significantly suppressed in MG2 patients compared with MG1 patients by more than 60% on average (**Figure 6C**).

PCA and blinded clustering of the plasma samples or patient data sets with average values of the plasma factors from the severe group also defined two patient groups, SG1 (*n*=13, 40.6%) and SG2 (*n*=19, 59.4%) (**Figures 6B, D**, and **Supplementary Figure S10**). The average z-score distribution of plasma factors in SG2 patients was significantly higher than that in SG1 patients, indicating more robust inflammatory responses in SG2 patients than in SG1 patients (**Figure 6E**). It is notable that the average z-score distribution in MG2 patients was even higher than that of SG1 patients, indicating that a proportion of moderate patients (MG2) could present even higher inflammatory responses than SG1 patients despite their lower clinical severity (**Figure 6E**). Nevertheless, we observed no significant difference in disease severity (**Figure 6F**) and patient age between the two severe patient groups (**Figure 6G**). A comparison of the mean values between SG1 and SG2 patients revealed that 102 plasma factors were significantly differentially regulated (85 factors upregulated and 17 factors downregulated in SG2 patients) (**Supplementary Table S5**). Among them, CXCL10 (4.3-fold), VWF (4.2-fold), PTX3 (3.7-fold), EGF (3.6-fold), CSF1 (3.6-fold), C5 (3.6-fold), REG3A (3.3-fold), CCL22 (3.3-fold), and IFNA1 (3.1-fold) in SG2 patients compared to SG1 patients were upregulated by more than three times on average. In contrast, IL12p40 (0.1-fold), THPO (0.2-fold), IFNG (0.3-fold), CX3CL1 (0.3-fold), KIT (0.3-fold), MMP12 (0.4-fold), and MICA (0.4-fold) were significantly suppressed in SG2 patients compared with SG1 patients by more than 60% on average (**Figure 6D**). Nonetheless, 14 factors, namely, IL18, WFDC2, HGF, VCAM1, ANGPT2, GDF15, IL1RL1, MUC16, TGFA, CCL23, MMP7, SPP1, PLAUR, and LGALS3, were consistently and significantly elevated in the severe patients (SG1 and SG2) compared with the moderate patients (MG1 and MG2) (**Supplementary Table S5**). Notably, the majority of these factors were mainly derived from respiratory epithelial cells (IL18, WFDC2, GDF15, MUC16, TGFA, MMP7, and LGALS3) and macrophages (IL18, SPP1, PLAUR, CCL23, and LGALS3) (**Figure 3C**; **Supplementary Figure S5C , S6C**).

## Discussion

The main target of SARS-CoV-2 in gas exchange units is type II alveolar cells, which serve as progenitor cells for type I cells and provide homeostatic repair mechanisms after injury. Hence, direct viral damage to type II cells can significantly impair respiratory function, often leading to severe pneumonic progression (26). Higher viral loads may not be critically associated with severe disease, as demonstrated by our current and previous studies (13, 27). Moreover, when we compared viral loads and kinetics in respiratory samples from our COVID-19 cohort and more pathogenic MERS-CoV-infected patients (mortality: 20.4%) during the 2015 Korean outbreak (**Supplementary Figure S1D, E**) (28), viral loads of SARS-CoV-2 in respiratory specimens generally peaked upon symptom onset and rapidly declined thereafter, whereas MERS-CoV loads peaked 4-10 days after symptom onset. There was no significant difference in overall viral loads and kinetics in COVID-19 patients depending on age and disease severity, but the viral loads of MERS-CoV were significantly higher among non-survivors than among survivors. Viral kinetics display more persistent replication with a clear delay in the peak response approximately 12-14 days after symptom onset in deceased MERS patients with older age (**Supplementary Figure S1D, E**) (28). Viral kinetics in pathogenic SARS-CoV-1 infections (mortality: ~10%) also show a peak response approximately 10 days after symptom onset (29). These results clearly indicate that more pathogenic CoVs, such as SARS-CoV-1 and MERS-CoV, present more sustained viral replication in the respiratory tracts of severe patients, with peak responses at approximately the second week after symptom onset, and the higher viral loads tend to be correlated with disease severity and patient age. In contrast, viral loads of SARS-CoV-2 peaking upon symptom onset declined rapidly as the disease and inflammation progressed regardless of disease severity and patient age, strongly suggesting that the degree of pathogenic inflammatory response seems to be determined primarily by host factors rather than higher viral loads.

Although previous studies have already reported massive proteomic analysis in sera or plasma from COVID-19 patients by mass spectrometry or proximity extension assays (16, 17, 30–34), our current study used highly sensitive and multiplexed immunoassays to measure precise concentration ranges in plasma. In addition, our cohort included a relatively large patient size, focused only on pneumonic patients with well-defined clinical scores and stages, and analyzed the majority of inflammatory factors associated with pulmonary and systemic inflammation reported in previous studies (14–19). In differential expression analysis between the moderate and severe COVID-19 groups, 169 factors out of 191 proteins were significantly upregulated (145 factors) or downregulated (24 factors) in plasma from severe patients compared to moderate patients (**Figure 1C**). These include a type I interferon, IFNA1, mainly derived from activated macrophages, and various inflammatory features of hyperinflammatory responses (IL6, IL10, IL13, MPO, and LBP), chemokine responses (CXCL10, CXCL9, CXCL11, IL-8, CCL4, CXCL13, CXCL2, CCL11, CXCL6, CCL2, CCL26, CCL19, CCL18, CCL24, CCL14, PF4, and CCL23), endothelial activation and coagulation (THBD, F3, VWF, PROS1, and MMP8), T-cell homeostasis and activation (IL7, IL15, IL18, IL4, and IL23A), and humoral responses (IL4R, IL1RL1, IL33, TNFSF13B, and C9) as well as elevated tissue damage responses, including vascular development (HGF, ENPP2, CHI3L1, THBS2, and ANGPT2), extracellular matrix organization (MMP2, MMP3, MMP9, PTX3, and SPP1), and epithelial mesenchymal transition (TGFB1, PLAUR, IGFBP2, IGFBP4, and SDC1). These results suggest the complexity and acute initiation of dysregulated systemic inflammation induced by SARS-CoV-2 infection in patients with severe COVID-19. In addition, our extensive correlation analysis of the plasma factors together with scRNA sequencing data from lung autopsy and respiratory samples further defined two core inflammatory modules, cores I and II, and their primary cellular sources (**Figure 3C**). The core I components were mainly contributed by activated macrophages, neutrophils, epithelial cells, and endothelial cells, whereas the core II network was primarily linked to activated epithelial cells, NK(T) cells, and T cells. The quantity and quality of correlation power tended to be increased in the core I and core II networks in a severity-dependent manner (**Figure 4**), suggesting enhanced complexity and functional interactions among leukocytes and pulmonary parenchymal cells as viral pneumonia progresses (1, 2, 17, 25, 35). Considering the enhanced infiltration of neutrophils, macrophages, and NKT cells into inflamed lung tissue but reduced NK- and T-cell responses in severe COVID-19 patients compared with moderate patients (**Supplementary Figure S5B, S6B**), our current data implied a differential role of innate and adaptive immune cells in pathological changes during disease progression *via* specific expression and interactions among the key players of both core modules. Of note, a substantial increase in the correlation power and expression level of the core II network members involved in NKT-cell homeostasis and effector function promoting both humoral and cell-mediated immunity (36–38) may support the pathogenic role of the NKT-cell population in severe inflammation, although the specific contribution of their subpopulations to COVID-19 is still controversial and needs to be verified (39). Moreover, the core I and II modules were further associated with complement activation (5, 40) and aberrant TGFB response (41), respectively, observed in severe COVID-19 patients. Several inflammatory mediators, such as ENPP2, C5, FASLG, VWF, and CSF1, additionally presented a more robust correlation with the core inflammatory networks in the severe group (**Figure 4C**), and these factors were reported as independent significant indicators of severe COVID-19 in previous studies (1, 5, 42–44). We also observed that the plasma levels of several proteins mainly derived from respiratory epithelial cells (IL18, WFDC2, GDF15, MUC16, TGFA, MMP7, and LGALS3) and macrophages (IL18, SPP1, PLAUR, CCL23, and LGALS3) were capable of distinguishing moderate and severe COVID-19 significantly and consistently regardless of the overall inflammatory status of the patients (**Supplementary Table S5**); hence, the damaging response of pulmonary epithelial cells and dysregulated macrophage activation might be critical determinants of severe pneumonic progression.

Interestingly, we noticed that IL12p40 and CX3CL1 displayed strong positive correlations with each other but showed robust negative correlations with several core inflammatory modules, mainly in the severe patient group (**Figure 4C**). In addition, molecular signatures of cell-mediated immunity, such as IL12p40, IFNG, and GZMB, in the M5 group were specifically and significantly higher than those in the NC, M4, and severe groups (**Figure 1C** and 2C), suggesting their protective and pathogenic roles in moderate pneumonia. An elevated IFNA1 response displayed a strong negative correlation with IL12p40 and CX3CL1, whereas it presented a robust positive correlation with CCL11, FASLG, and IL23A, especially in severe COVID-19 patients (**Figure 5**). This also suggested that an aberrant type I IFN response during the acute phase of SARS-CoV-2 infection may be associated with immune dysregulation and disease progression in severe COVID-19 patients requiring extensive respiratory support. Indeed, our current data using sensitive immunoassays of the plasma of pneumonic COVID-19 patients support the pathogenic role of an acute surge in the IFNA1 response, at least in a proportion of severe pneumonia patients and COVID-19 patients who died, even though the specific role of type I and III interferons in severe COVID-19 progression is still controversial (45, 46). One of the striking features is the heterogeneous phenotype of hyperinflammation associated with the strong inverse correlation of IL12p40 and IFNA1 observed in both moderate and severe COVID-19 pneumonia patients (**Figure 5** and 6). Given that IL12p40 is a common subunit of IL12p70 (heterodimer of IL12p35 and IL12p40) and IL23 (heterodimer of IL23p19 and IL12p40) (47), it is also intriguing to see significant upregulation of both IL12p70 and IL23 in severe patients than in moderate subjects (**Figure 1C**). In addition, IL12p40 presented significant negative correlation with both IL12p70 and IL23 (IL23p19) in COVID-19 patients (**Supplementary Figure S11**). Differential expression of these cytokine subunits may be due to heterogeneity of their primary cellular resources (IL12p40 from macrophages, IL12p35 and IL23p19 from non-hematopoietic epithelial cells or endothelial cells, see Figure S5 and S6) and heterodimeric interactions among various IL12 family cytokine subunits (47) during COVID-19 inflammation. Even though the heterodimeric subunits of IL-12 and IL23 are known to be simultaneously co-expressed in activated myeloid cells, they can also assemble to form functionally active heterodimers after secretion from different cell types *via* alternate two-cell pathway (48). Further study on regulation mechanisms governing the differential expression of various IL12 family cytokine subunits might be required to define their specific role in COVID-19 inflammation. Nonetheless, enhanced IFNA1 and a suppressed IL12p40 response strongly associated with persistent and overt inflammatory responses were detected even in a proportion of moderate pneumonia patients (8.3%, MG2) and in more than half (59.4%, SG2) of severe COVID-19 patients. Therefore, this unexpected reciprocal correlation of IFNA1 and IL12p40, mainly expressed in macrophages (**Figure 4C**), might also be strongly associated with the heterogeneity of dysregulated inflammation in pneumonic COVID-19 patients. Given that the severity of illness during acute COVID-19 is significantly but partially associated with long COVID-19 syndrome or post-acute COVID-19 (49), further follow-up studies on the potential linkage of post-acute COVID-19 with the overt inflammatory response associated with an inverse correlation of higher IFNA1 and lower IL12p40 are needed. A recent review on long COVID-19 after breakthrough SARS-CoV-2 infection also suggested that vaccination before infection confers only partial protection in the post-acute phase of the disease and emphasized the need for continued optimization of strategies against post-acute syndrome even for people with breakthrough infection (50).

A limitation of our study is the inclusion of plasma samples rather biased toward moderate pneumonia cases (sample *n*=745) than severe pneumonia cases (sample *n*=130), and all these samples were obtained from unvaccinated patients with primary SARS-CoV-2 infection. The patients were also treated with various combinations of antiviral drugs and corticosteroids depending on disease severity during hospitalization. Therefore, further validation with a larger scale of plasma specimens from clinically variable COVID-19 patients, even after vaccination or reinfection, is needed.

## Data availability statement

The datasets presented in this study can be found in online repositories. The names of the repository/repositories and accession number(s) can be found in the article/**Supplementary Material**.

## Ethics statement

The clinical research was approved by the institutional review boards of Chungnam National University Hospital (IRB no.: CNUH 2020-12-002-008), Seoul Medical Center (IRB no.: SEOUL 2021-02-016), Samsung Medical Center (IRB no.: SMC-2021-03-160), Seoul National University Hospital (IRB no.: C-1509-103-705), and the Korea National Institute of Health (IRB no.: 2020-09-03-C-A). The patients/participants provided their written informed consent to participate in this study.

## Author contributions

W-YP, Y-SK, H-YP and N-HC designed this research. KJ, YK, SK, UP, JK, NP, JK, N-YH, M-SS, MK, YR, HJ, SL, DP, JL, HK, SK, J-HL, JS, HK, YJ, MC, H-YP, W-YP, Y-SK and N-HC collect human specimens, performed experiments, and analyzed the datasets. KJ, JayK, JS, HK, YJ, MC, and N-HC organized and presented the results. KJ, YK, UP, JaeK, YJ, MC, and N-HC contributed to the writing of the draft. JayK, HK, YJ, Y-SC, HK, W-WL, MC, and N-HC reviewed and revised the manuscript. All authors listed have made a substantial, direct, and intellectual contribution to the work and approved it for publication.
