# Corrigendum: Elevated IFNA1 and suppressed IL12p40 associated with persistent hyperinflammation in COVID-19 pneumonia

**DOI:** 10.3389/fimmu.2023.1175767

**Published:** 2023-03-08

**Authors:** Kyeongseok Jeon, Yuri Kim, Shin Kwang Kang, Uni Park, Jayoun Kim, Nanhee Park, Jaemoon Koh, Man-Shik Shim, Minsoo Kim, Youn Ju Rhee, Hyeongseok Jeong, Siyoung Lee, Donghyun Park, Jinyoung Lim, Hyunsu Kim, Na-Young Ha, Hye-Yeong Jo, Sang Cheol Kim, Ju-Hee Lee, Jiwon Shon, Hoon Kim, Yoon Kyung Jeon, Youn-Soo Choi, Hye Young Kim, Won-Woo Lee, Murim Choi, Hyun-Young Park, Woong-Yang Park, Yeon-Sook Kim, Nam-Hyuk Cho

**Affiliations:** ^1^ Department of Microbiology and Immunology, Seoul National University College of Medicine, Seoul, Republic of Korea; ^2^ Department of Biomedical Sciences, Seoul National University College of Medicine, Seoul, Republic of Korea; ^3^ Department of Thoracic and Cardiovascular Surgery, Chungnam National University School of Medicine, Deajon, Republic of Korea; ^4^ Medical Research Collaborating Center, Seoul National University Hospital, Seoul, Republic of Korea; ^5^ Department of Pathology, Seoul National University College of Medicine, Seoul, Republic of Korea; ^6^ Department of Internal Medicine, Chungnam National University School of Medicine, Deajon, Republic of Korea; ^7^ Geninus Inc., Seoul, Republic of Korea; ^8^ Samsung Genome Institute, Samsung Medical Center, Seoul, Republic of Korea; ^9^ Chungnam National University Hospital, Biomedical Research Institute, Deajon, Republic of Korea; ^10^ Division of Healthcare and Artificial Intelligence, Department of Precision Medicine, Korea National Institute of Health, Korea Disease Control and Prevention Agency, Cheongju, Republic of Korea; ^11^ Department of Biohealth Regulatory Science, School of Pharmacy, Sungkyunkwan University, Suwon-si, Gyeonggi-do, Republic of Korea; ^12^ Biopharmaceutical Convergence Major, School of Pharmacy, Sungkyunkwan University, Suwon-si, Gyeonggi-do, Republic of Korea; ^13^ Department of Precision Medicine, Korea National Institute of Health, Korea Disease Control and Prevention Agency, Cheongju, Republic of Korea; ^14^ Institute of Endemic Diseases, Medical Research Center, Seoul National University, Seoul, Republic of Korea; ^15^ Seoul National University Bundang Hospital, Seongnam, Gyeonggi-do, Republic of Korea; ^16^ Wide River Institute of Immunology, Seoul National University, Hongcheon, Gangwon-do, Republic of Korea

**Keywords:** COVID-19, SARS-CoV-2, pneumonia, inflammation, IFNa, IL-12p40

In the published article, there was a mistake in the Funding statement. The grant number for the National Research Foundation (NRF) funded by the Ministry of Science and ICT was displayed as “2021M3A9H5020761”. The correct Funding statement appears below.

This work was supported by grants from the Korea National Institute of Health Infrastructural Research Program (4800–4861–312–210–13), operation of data center for national biomedical data resources (2021-NI-017-00), the National Research Foundation (NRF) funded by the Ministry of Science and ICT (2021M3A9I2080490), 2022 Joint Research Project of Institutes of Science and Technology (to N-HC), and the Basic Science Research Program through the National Research Foundation of Korea funded by the Ministry of Education (2022R1A6A3A1307317). KJ and UP received a scholarship from the BK21-plus education program provided by the National Research Foundation of Korea.

The authors apologize for this error and state that this does not change the scientific conclusions of the article in any way. The original article has been updated.

