# Supplementary material for: Elevated IFNA1 and suppressed IL12p40 associated with persistent hyperinflammation in COVID-19 pneumonia
Source: Front Immunol. 2023 Jan 27;14:1101808. doi: 10.3389/fimmu.2023.1101808 (PMC9911526; doi:10.3389/fimmu.2023.1101808)
Supplement: Supplementary file 1 [file DataSheet_1.pdf]

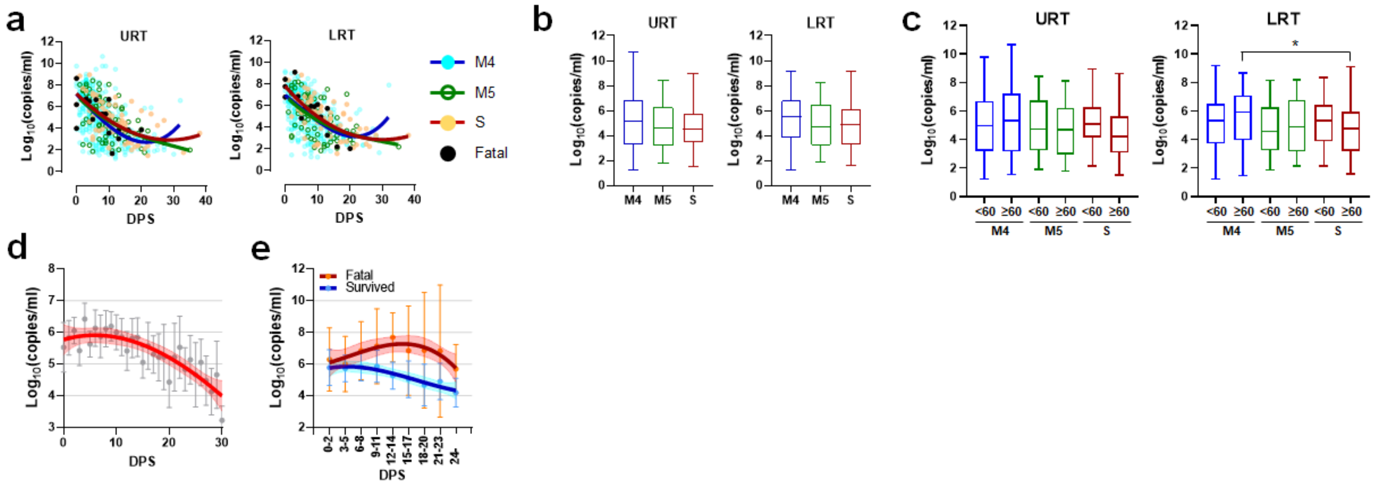

Figure S1. Kinetic changes and overall viral loads in the upper (URT) and lower (LRT) respiratory tract of COVID-19 patients. **A.** Kinetic changes in viral loads in the URT (M4, n=361; M5, n=72; S, n=85) or LRT (M4, n=340; M5, n=75; S, n=88) samples from the indicated COVID-19 patient groups are presented. The colored lines show the trend in viral loads according to disease severity, using curve fit with nonlinear regression. DPS, days post-symptom onset. **B** and **C.** Distribution of viral loads in the URT and LRT samples from the indicated severity groups (**B**) and age groups (**C**) are presented as a box and whisker (min to max) plot with median line. *, $p < 0.05$ (Kruskal–Wallis test). **D** and **E**. Kinetic changes in viral loads in sputum samples (n=1,714) from 185 Korean MERS patients (**D**) and comparison of viral kinetics in surviving (n=147) and deceased patients (n=38) (**E**). Viral load data from MERS patients were adopted from ref. 28.

A

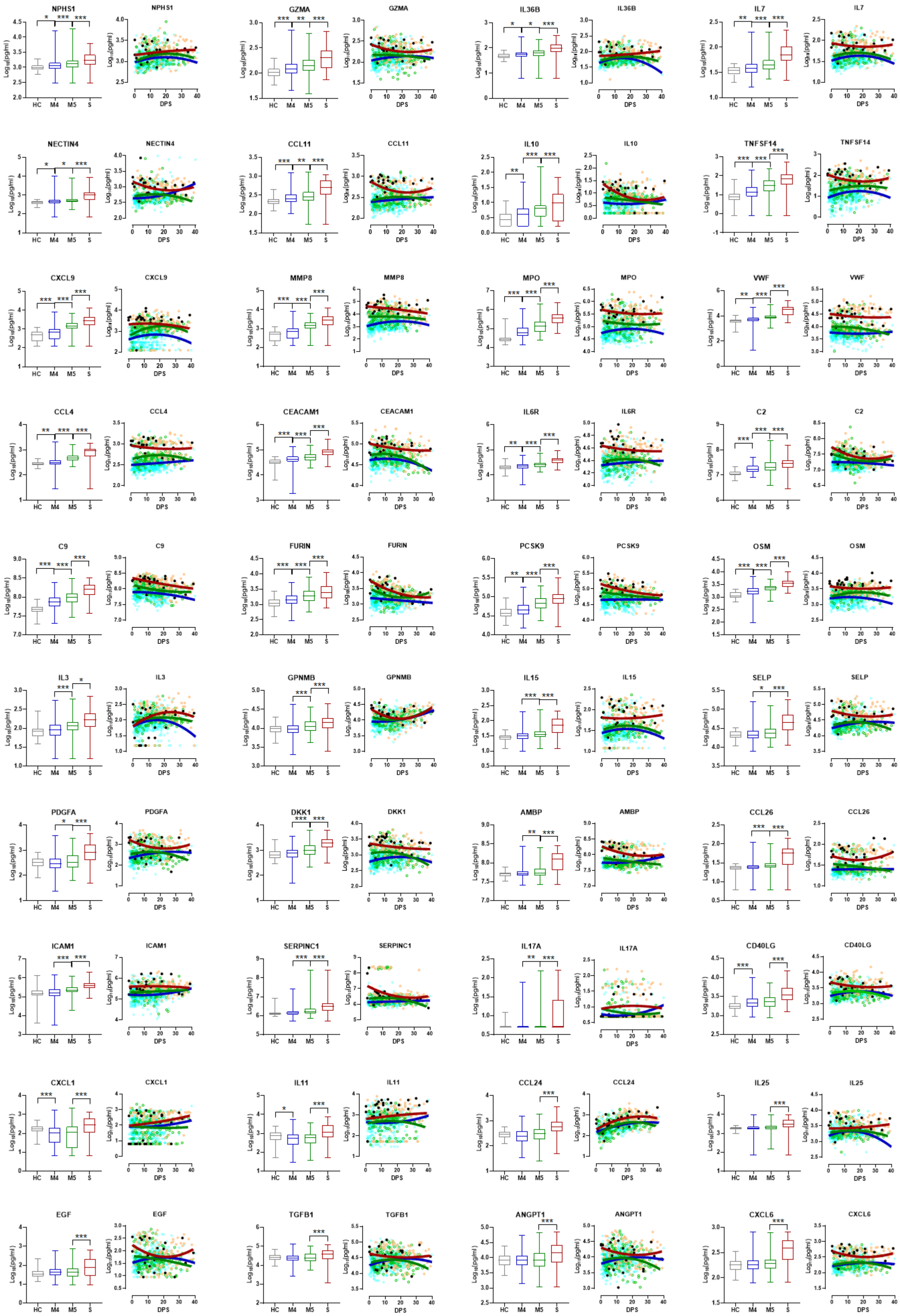

NPHS1
GZMA
IL36B
IL7
NECTIN4
CCL11
IL10
TNFSF14
CXCL9
MMP8
MPO
VWF
CCL4
CEACAM1
IL6R
C2
C9
FURIN
PCSK9
OSM
IL3
GPNMB
IL15
SELP
PDGFA
DKK1
AMBP
CCL26
ICAM1
SERPINC1
IL17A
CD40LG
CXCL1
IL11
CCL24
IL25
EGF
TGFB1
ANGPT1
CXCL6
Log10(pg/ml)
HC
M4
M5
S
DPS

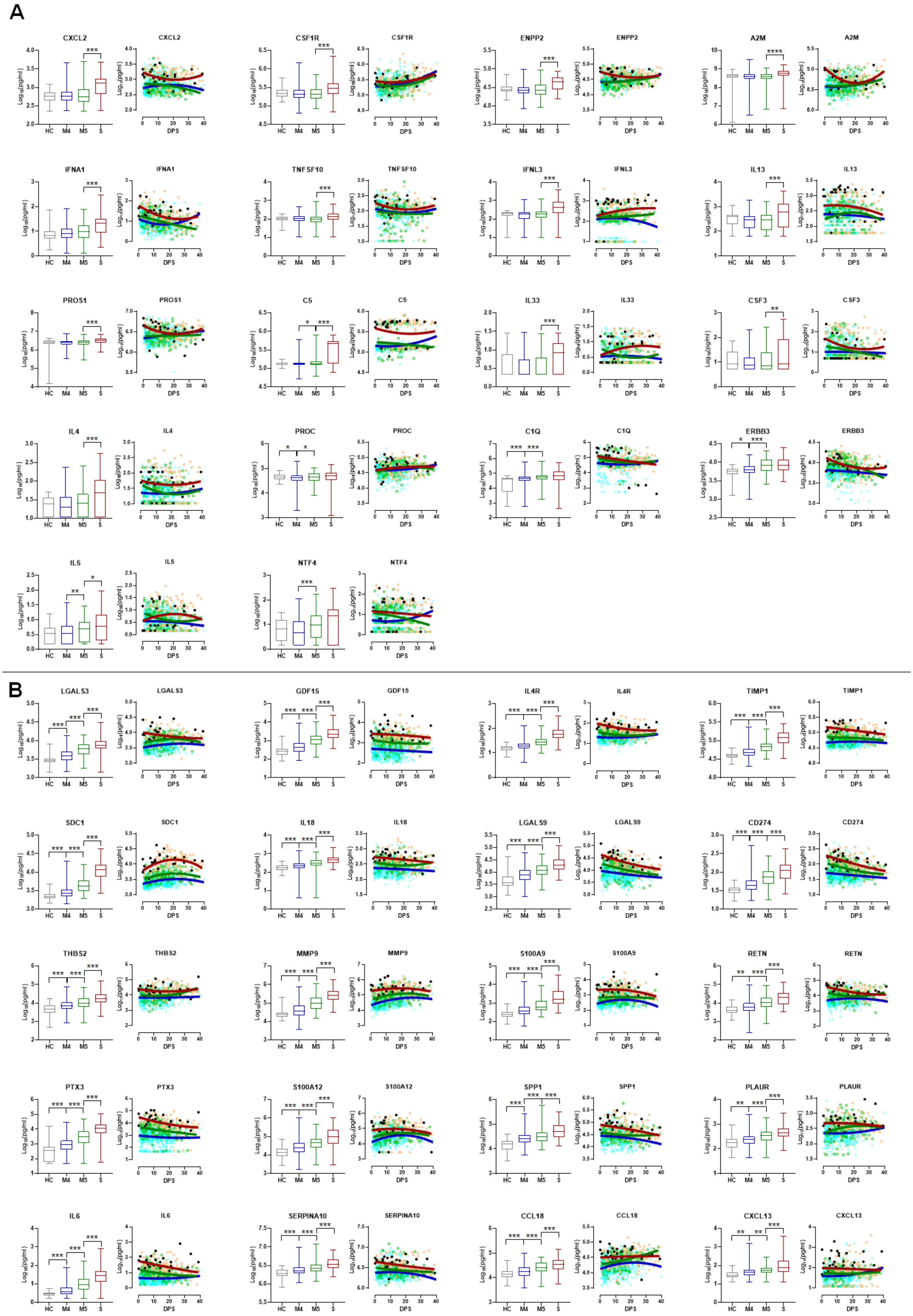
A
CXCL2
CSF1R
ENPP2
A2M
IFNA1
TNFSF10
IFNL3
IL13
PROS1
C5
IL33
CSF3
IL4
PROC
C1Q
ERBB3
IL5
NTF4
B
LGALS3
GDF15
IL4R
TIMP1
SDC1
IL18
LGALS9
CD274
THBS2
MMP9
S100A9
RETN
PTX3
S100A12
SPP1
PLAUR
IL6
SERPINA10
CCL18
CXCL13
HC
M4
M5
S
DPS
Log10(pg/ml)

B

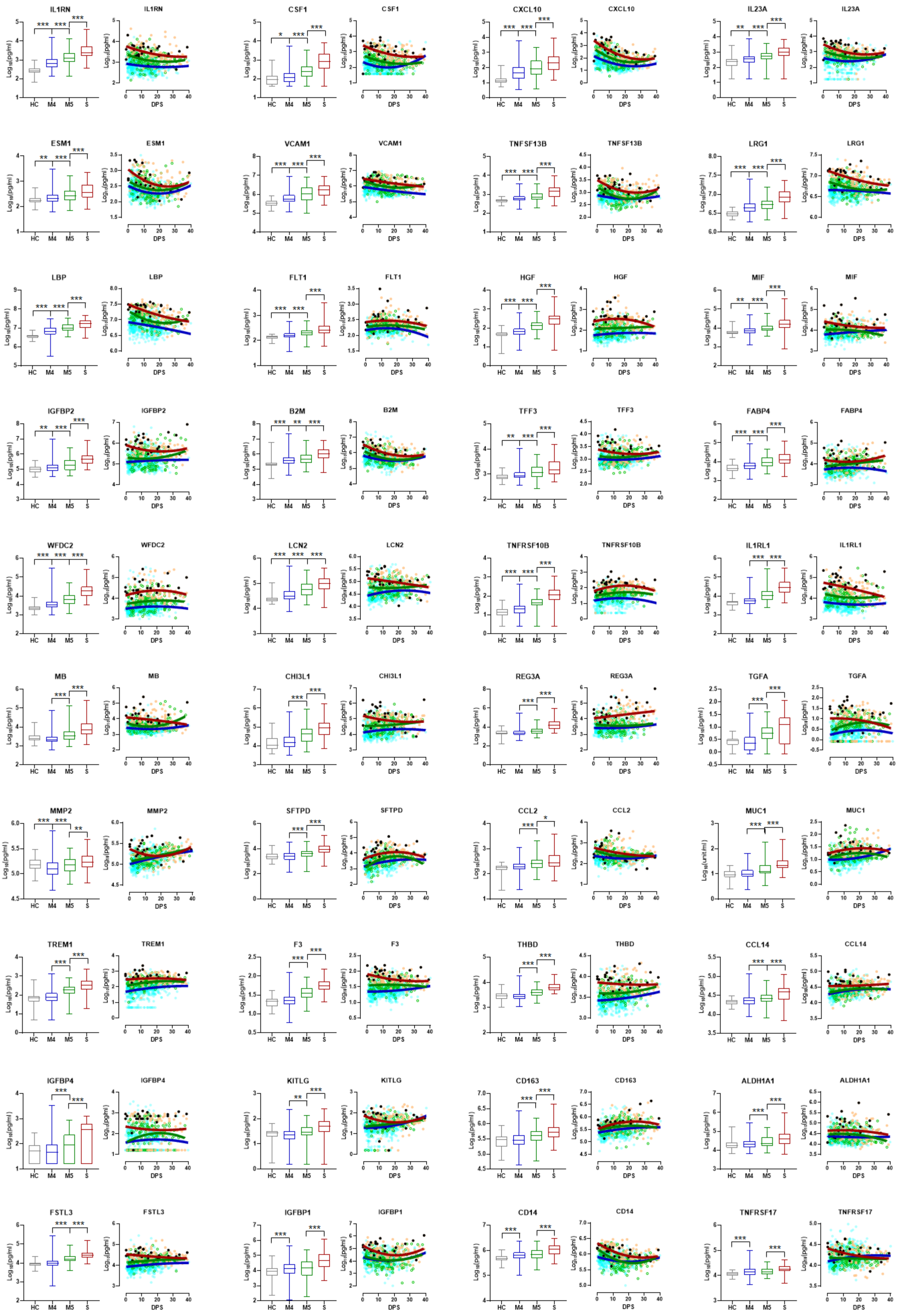
IL1RN
CSF1
CXCL10
IL23A
ESM1
VCAM1
TNFSF13B
LRG1
LBP
FLT1
HGF
MIF
IGFBP2
B2M
TFF3
FABP4
WFDC2
LCN2
TNFRSF10B
IL1RL1
MB
CHI3L1
REG3A
TGFA
MMP2
SFTPD
CCL2
MUC1
TREM1
F3
THBD
CCL14
IGFBP4
KITLG
CD163
ALDH1A1
FSTL3
IGFBP1
CD14
TNFRSF17
HC
M4
M5
S
DPS
Log_{10}(pg/ml)
Log_{10}(unit/ml)

B

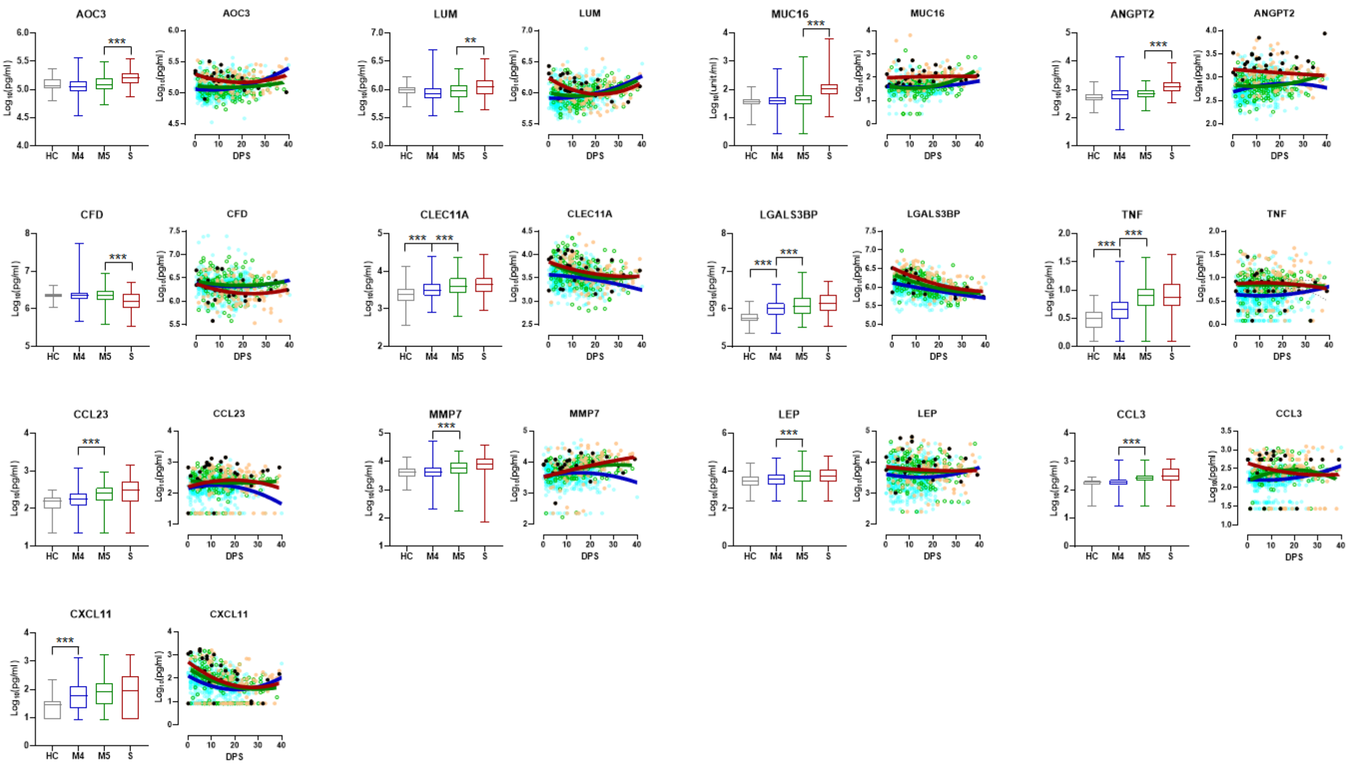
AOC3
LUM
MUC16
ANGPT2
CFD
CLEC11A
LGALS3BP
TNF
CCL23
MMP7
LEP
CCL3
CXCL11
HC
M4
M5
S
DPS
Log10(pg/ml)

C

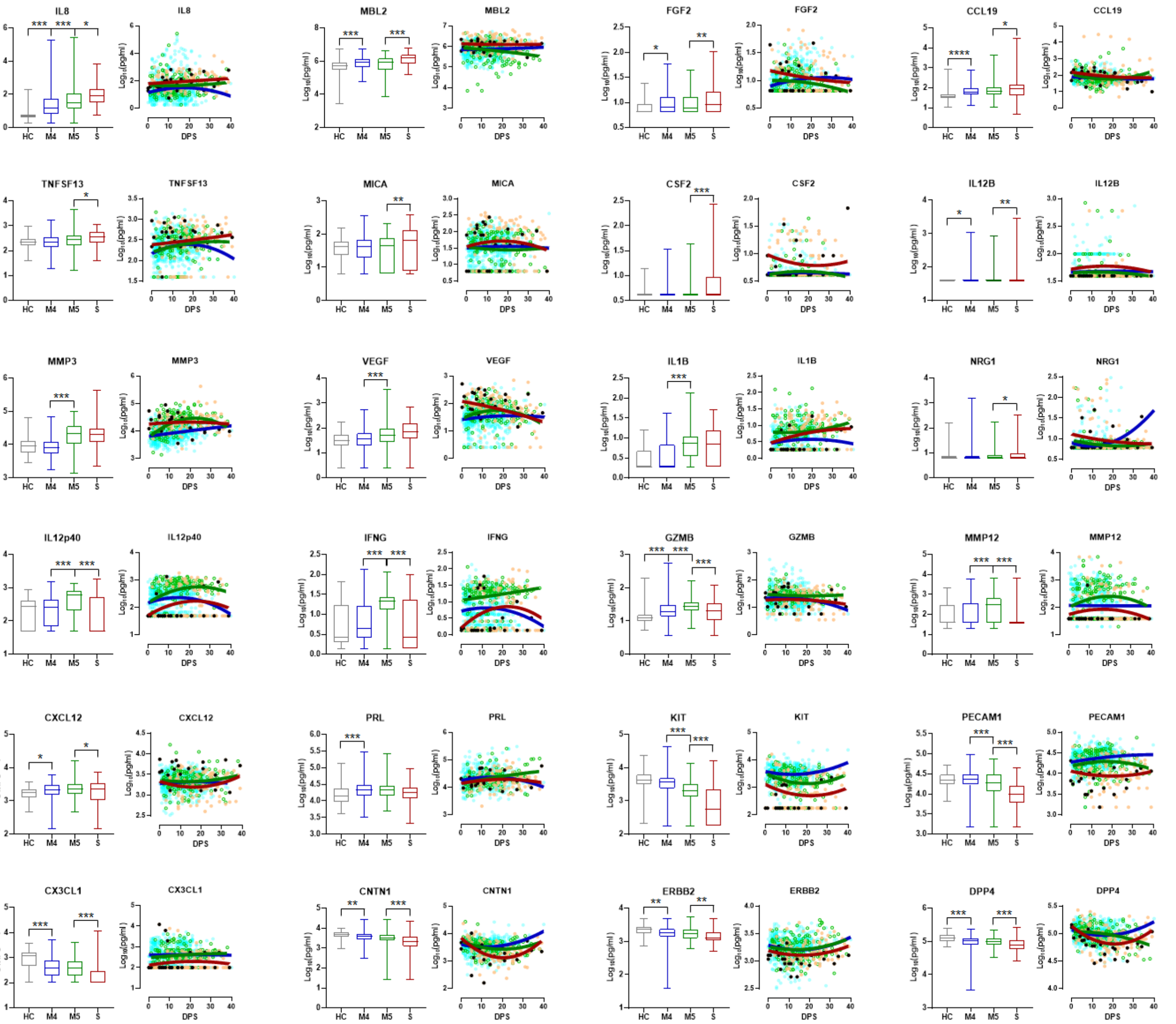
IL8
MBL2
FGF2
CCL19
TNFSF13
MICA
CSF2
IL12B
MMP3
VEGF
IL1B
NRG1
IL12p40
IFNG
GZMB
MMP12
CXCL12
PRL
KIT
PECAM1
CX3CL1
CNTN1
ERBB2
DPP4
HC
M4
M5
S
DPS
Log10(pg/ml)

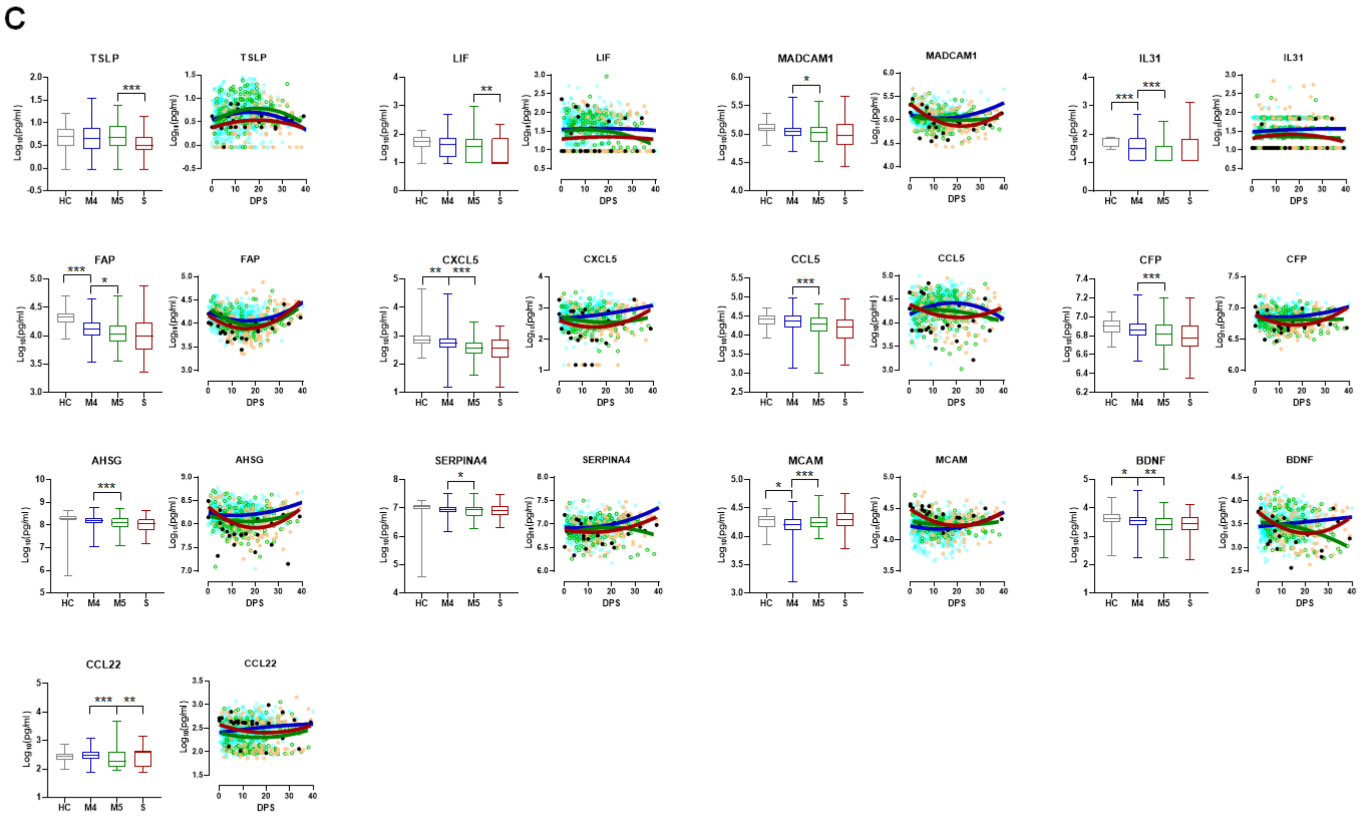

Figure S2. Kinetic responses of 170 plasma proteins showing significant differences among NC, M4, M5, and severe (S) groups. Kinetic changes and distribution of 170 plasma factor levels belonging to cluster 1 (C1, **A**), 2 (C2, **B**), 3, and 4 (C3 and C4, **C**) are presented. Solid lines indicate nonlinear regression. Gray: NC, blue: M4 group, green: M5 group, red: severe group, and black dots for fatal cases. *, $p < 0.05$; **, $p < 0.01$; ***, $p < 0.001$.

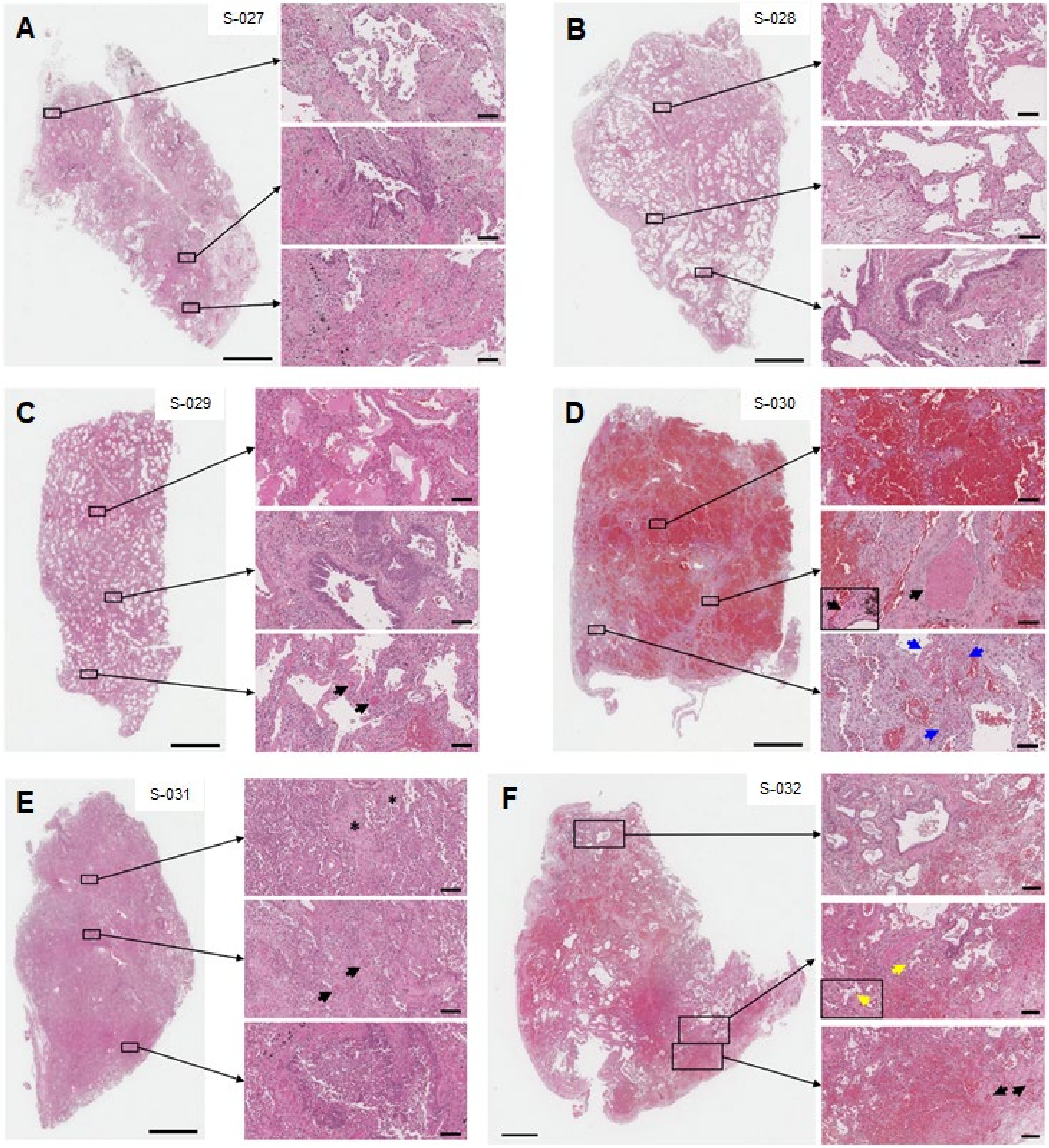

Figure S3. Histopathologic examination of lung autopsy samples from deceased COVID-19 patients after H&E staining. **A**. Diffuse subpleural and interstitial fibrosis are observed (left). Higher power views of each representative area of different anatomic sites (black squares) are displayed. Destroyed alveolar wall with fibrosis and epithelial hyperplasia are observed (right upper). Peribronchiolar lymphoplasma cell infiltration is observed (right middle). In addition, some lymphocytic infiltration and smooth muscle proliferation in dense fibrotic interstitium are observed (right lower). **B**. Diffuse alveolar wall thickening is observed (left). In thickened alveolar septal area, partially destroyed alveolar wall with type II pneumocyte hyperplasia and detachment and focal hyaline membrane formation along with some inflammatory cell infiltration are seen (right upper). Subpleural and interstitial fibrosis are locally observed (right middle). In bronchus, some lymphoplasmacytic infiltration is observed in the peribronchial area (right lower). **C**. Diffusely thickened alveolar septa and some eosinophilic materials and edema in alveolar spaces are observed at a scan view (left). Diffuse hyaline membranes deposit along the alveolar walls and intra-alveolar fibrinous material accumulation and edema are observed (right upper). Acute bronchitis is observed by identifying intraepithelial neutrophil infiltration (right middle). There are some microthrombi in small pulmonary

vessels (black arrows, right lower). **D**. Marked diffuse alveolar hemorrhage is noted (left). Congestion in alveolar space and thickening of the alveolar wall are observed (right upper). In addition, large vessel thrombus (black arrow) and microthrombi (insert, black arrow) in small pulmonary vessels are seen (right middle). In alveoli, fibrin deposits (blue arrows), edematous change and fibroblasts are observed (right lower). **E**. Diffuse irregularly thickened alveolar walls with collapsed alveolar space are observed (left). Mixed inflammatory cells, such as neutrophils, alveolar macrophages and a few lymphocytes in alveolar space and type II pneumocyte hyperplasia are observed. Fibroblastic proliferation with organization is observed (asterisks) (right upper). Multiple microthrombi in small pulmonary vessels are noted (black arrows, right middle). Acute bronchitis with inflammatory exudate is observed (right lower). **F**. Chronic inflammation in bronchus, denuded pneumocyte type II pneumocyte, and subpleural and interstitial fibrotic inflammation are observed. Interstitial fibrosis and bronchial metaplasia are detected (right upper). Interstitial fibrosis and bronchial metaplasia are observed (right middle). Type II pneumocyte hyperplasia is also observed (yellow arrow). Intra-alveolar and interstitial hemorrhage and microthrombi (black arrow) are detected (right lower).

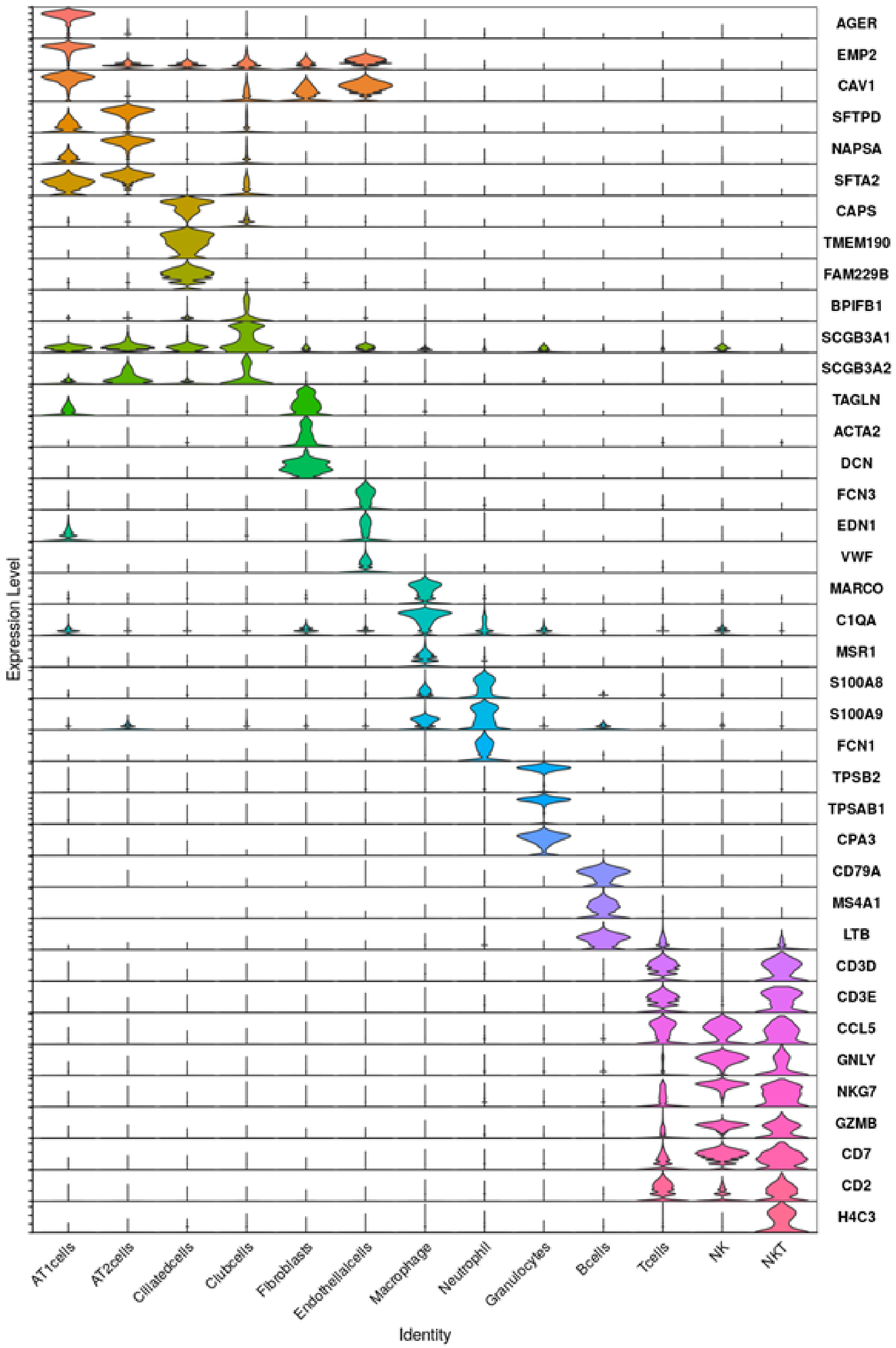

Figure S4. Violin plot for cell-type-specific signature genes. Genes were selected on the basis of the expression levels of the three most characteristic genes.

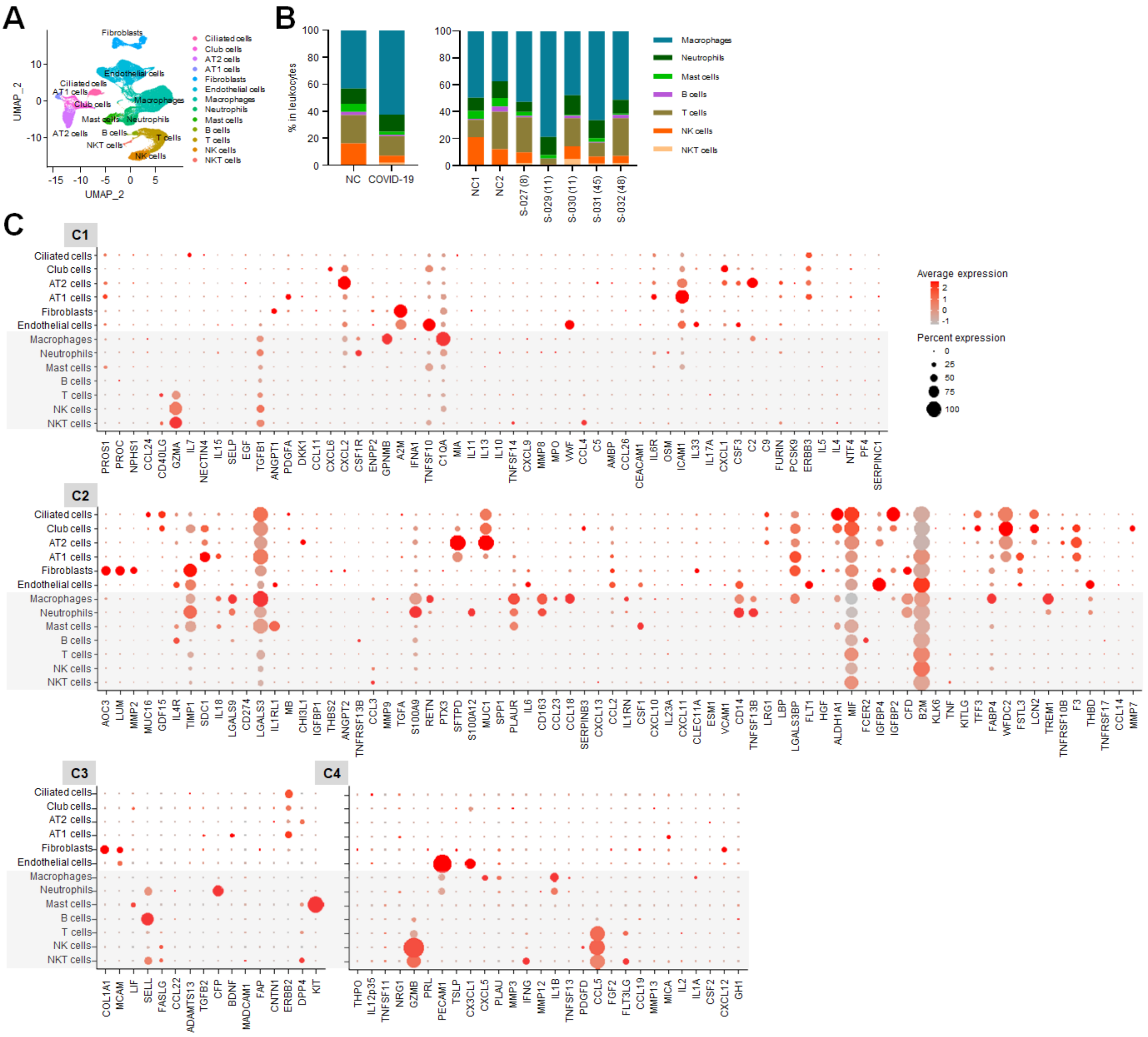

Figure S5. Primary cellular sources of 174 plasma factors identified by cellular transcript levels in scRNA sequencing data from lung autopsy. **A**. UMAP presentation of major cell types and associated clusters in respiratory leukocytes and parenchymal cells. **B**. Proportion bar plots of seven major leukocyte types in lung tissues from NCs (n=2) and deceased COVID-19 patients (n=5) (left). Right graph shows proportion of individual data from NCs and COVID-19 patients. **C**. Dot plot for 174 plasma factor transcripts level in respiratory leukocytes and parenchymal cells. Average and percent expression of transcript encoding the corresponding factor were scaled according to indices.

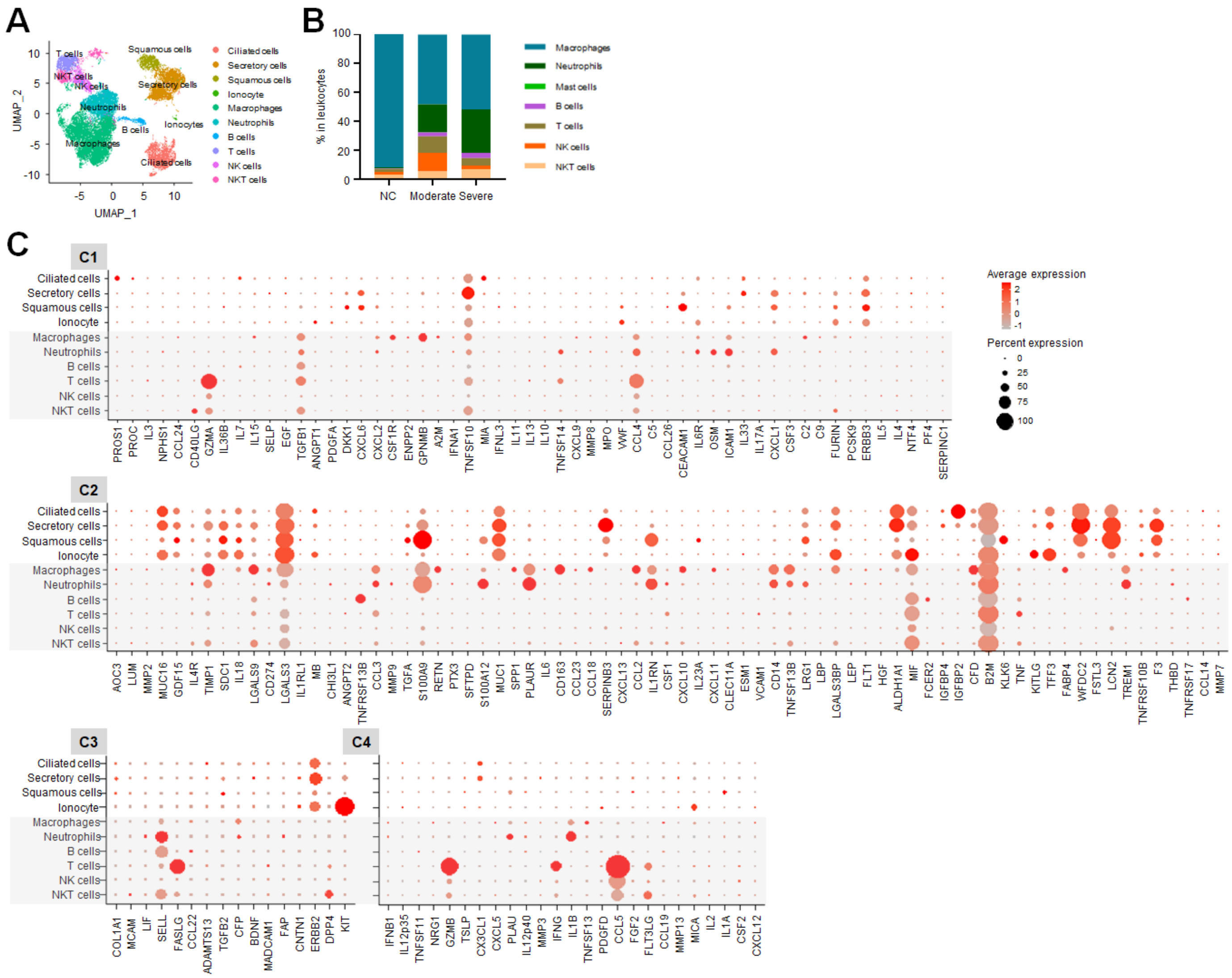

Figure S6. Primary cellular sources of 169 plasma factors identified by cellular transcript levels in scRNA sequencing data set from respiratory samples (ref. 8 and 41). **A**. UMAP presentation of major cell types and associated clusters in respiratory leukocytes and epithelial cells. **B**. Proportion bar plots of seven major leukocyte types in lung tissues from NCs (*n*=9) and moderate (*n*=11) and severe (*n*=17) COVID-19 patients. **C**. Dot plot for 169 plasma factor transcripts level in respiratory leukocytes and epithelial cells. Average and percent expression of transcript encoding the corresponding factor were scaled according to indices.

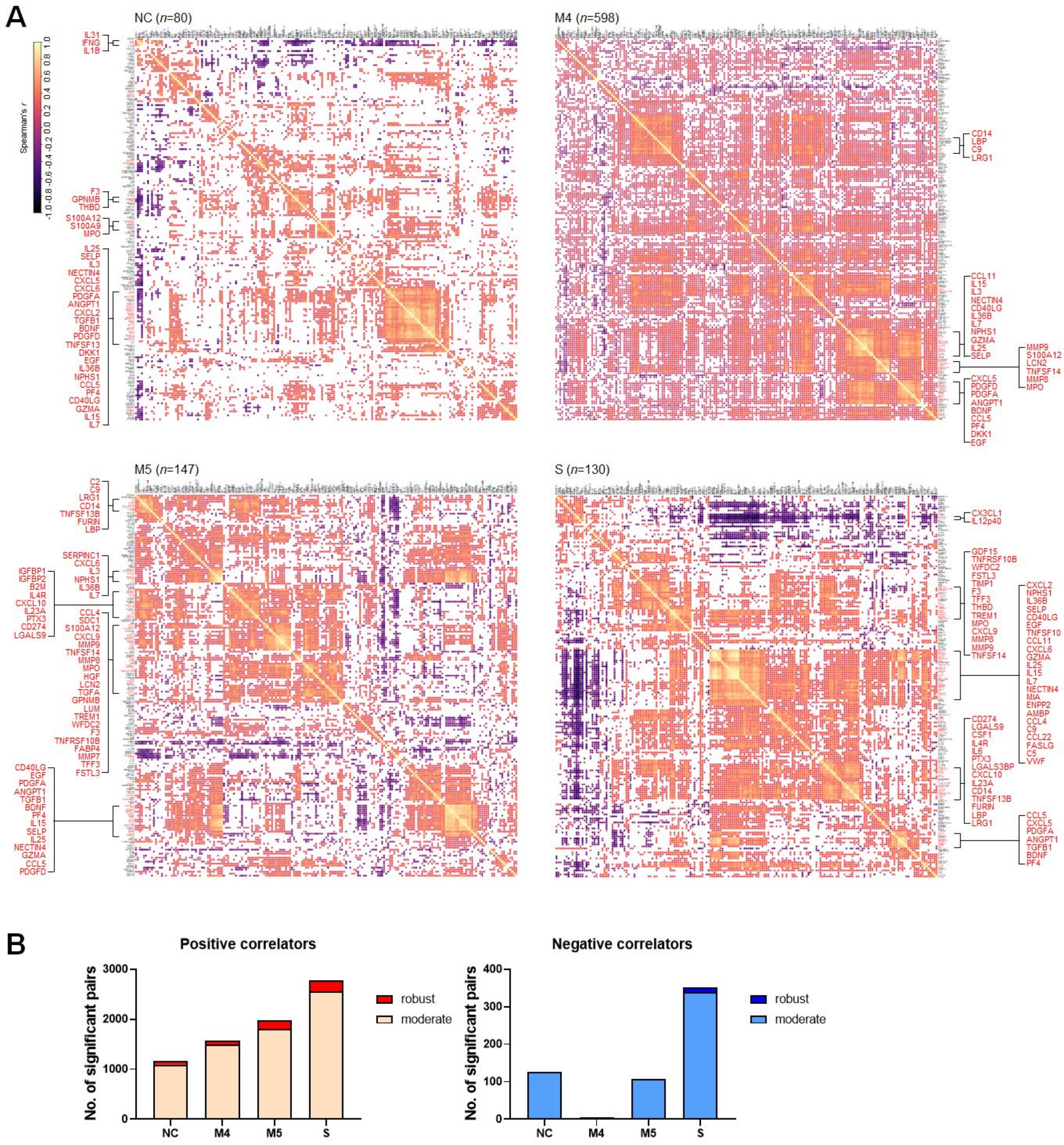

Figure S7. Enhanced correlation in quantity and quality among the plasma factors according to COVID-19 severity. **A**, Correlation matrix across all time points of 191 plasma factors from NCs and COVID-19 patients (M4 and M5, moderate; S, severe). Only significant correlations ($p < 0.05$) are represented as dots. Spearman's correlation coefficients from comparisons of protein measurements within the same specimen are visualized by color intensity. Components with robust correlation (absolute Spearman's $r \geq 0.7$) are indicated by red characters. **B**. Distribution of the number of significant correlation pairs ($p < 0.05$). Red (robust positive correlation): Spearman's $r \geq 0.7$, orange (moderate positive correlation): $0.7 >$ Spearman's $r \geq 0.4$, blue (robust negative correlation): Spearman's $r \leq -0.7$, sky blue (moderate negative correlation): $-0.7 <$ Spearman's $r \leq -0.4$.

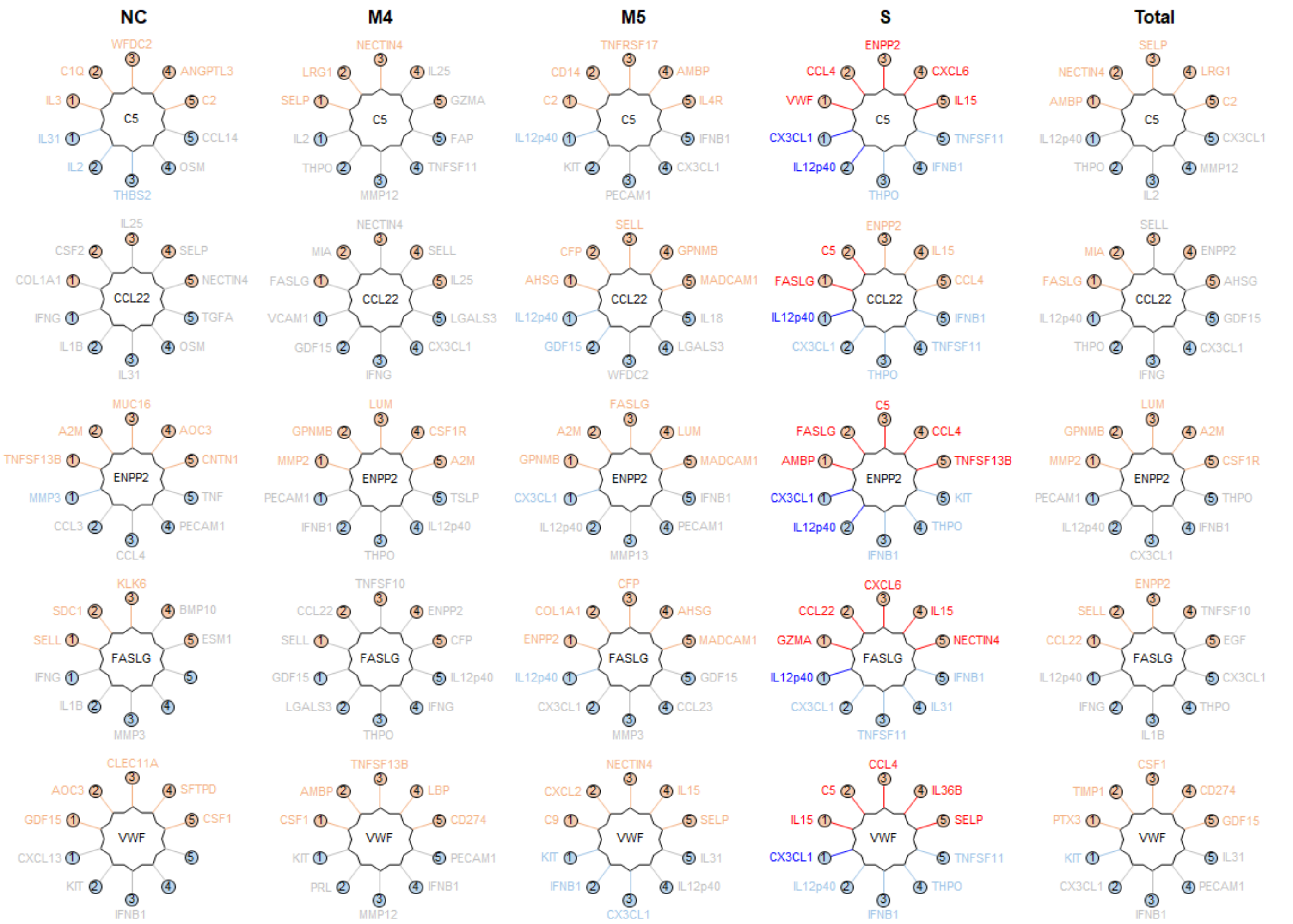

Figure S8. Robust inverse correlation of IL12p40 and CX3CL1 with inflammatory mediators specifically observed in severe COVID-19 patients. Top 5 positive and negative correlators of C5, CCL22, ENPP2, FASLG, and VWF in NCs and COVID-19 patients with various disease severities (M4 and M5: moderate pneumonia, S: severe pneumonia). Upper factors are the best 5 positive correlators and lower mediators are the best 5 negative correlators of the corresponding protein. The color of each factor is annotated according to the value of their correlation coefficient; red and blue: robust correlation with absolute Spearman's $r \geq 0.7$, orange and sky blue: moderate correlation with $0.7 >$ absolute Spearman's $r \geq 0.4$, and gray: no significant correlation ($p > 0.05$).

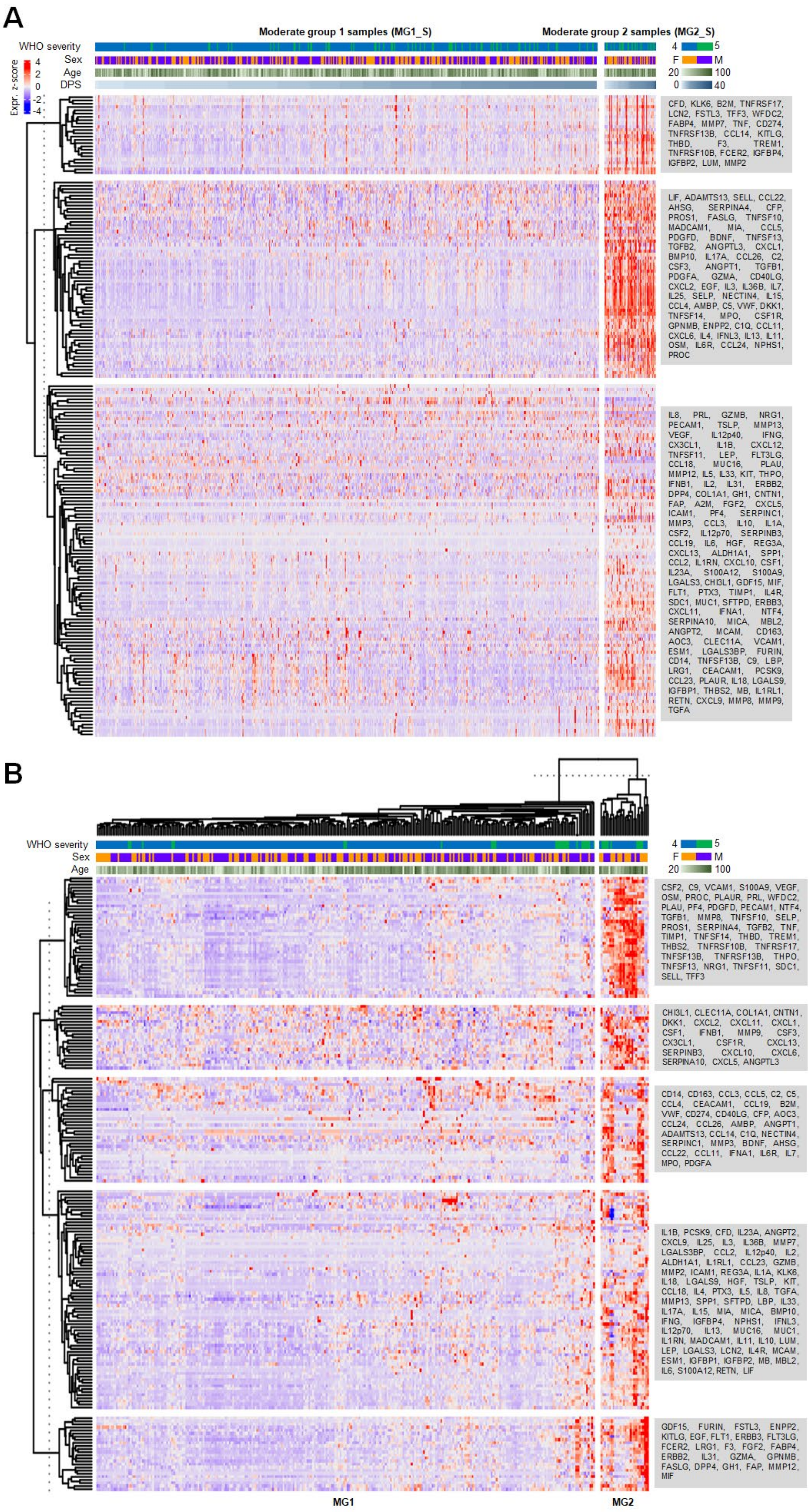

A
Moderate group 1 samples (MG1_S)
Moderate group 2 samples (MG2_S)
WHO severity
Sex
Age
DPS
Expr. z-score
4
2
0
-2
-4
4 5
F M
20 100
0 40
CFD, KLK6, B2M, TNFRSF17, LCN2, FSTL3, TFF3, WFDC2, FABP4, MMP7, TNF, CD274, TNFRSF13B, CCL14, KITLG, THBD, F3, TREM1, TNFRSF10B, FCER2, IGFBP4, IGFBP2, LUM, MMP2
LIF, ADAMTS13, SELL, CCL22, AHSG, SERPINA4, CFP, PROS1, FASLG, TNFSF10, MADCAM1, MIA, CCL5, PDGFD, BDNF, TNFSF13, TGFB2, ANGPTL3, CXCL1, BMP10, IL17A, CCL26, C2, CSF3, ANGPT1, TGFB1, PDGFA, GZMA, CD40LG, CXCL2, EGF, IL3, IL36B, IL7, IL25, SELP, NECTIN4, IL15, CCL4, AMBP, C5, VWF, DKK1, TNFSF14, MPO, CSF1R, GPNMB, ENPP2, C1Q, CCL11, CXCL6, IL4, IFNL3, IL13, IL11, OSM, IL6R, CCL24, NPHS1, PROC
IL8, PRL, GZMB, NRG1, PECAM1, TSLP, MMP13, VEGF, IL12p40, IFNG, CX3CL1, IL1B, CXCL12, TNFSF11, LEP, FLT3LG, CCL18, MUC16, PLAU, MMP12, IL5, IL33, KIT, THPO, IFNB1, IL2, IL31, ERBB2, DPP4, COL1A1, GH1, CNTN1, FAP, A2M, FGF2, CXCL5, ICAM1, PF4, SERPINC1, MMP3, CCL3, IL10, IL1A, CSF2, IL12p70, SERPINB3, CCL19, IL6, HGF, REG3A, CXCL13, ALDH1A1, SPP1, CCL2, IL1RN, CXCL10, CSF1, IL23A, S100A12, S100A9, LGALS3, CHI3L1, GDF15, MIF, FLT1, PTX3, TIMP1, IL4R, SDC1, MUC1, SFTPD, ERBB3, CXCL11, IFNA1, NTF4, SERPINA10, MICA, MBL2, ANGPT2, MCAM, CD163, AOC3, CLEC11A, VCAM1, ESM1, LGALS3BP, FURIN, CD14, TNFSF13B, C9, LBP, LRG1, CEACAM1, PCSK9, CCL23, PLAUR, IL18, LGALS9, IGFBP1, THBS2, MB, IL1RL1, RETN, CXCL9, MMP8, MMP9, TGFA
B
WHO severity
Sex
Age
4 5
F M
20 100
CSF2, C9, VCAM1, S100A9, VEGF, OSM, PROC, PLAUR, PRL, WFDC2, PLAU, PF4, PDGFD, PECAM1, NTF4, TGFB1, MMP8, TNFSF10, SELP, PROS1, SERPINA4, TGFB2, TNF, TIMP1, TNFSF14, THBD, TREM1, THBS2, TNFRSF10B, TNFRSF17, TNFSF13B, TNFRSF13B, THPO, TNFSF13, NRG1, TNFSF11, SDC1, SELL, TFF3
CHI3L1, CLEC11A, COL1A1, CNTN1, DKK1, CXCL2, CXCL11, CXCL1, CSF1, IFNB1, MMP9, CSF3, CX3CL1, CSF1R, CXCL13, SERPINB3, CXCL10, CXCL6, SERPINA10, CXCL5, ANGPTL3
CD14, CD163, CCL3, CCL5, C2, C5, CCL4, CEACAM1, CCL19, B2M, VWF, CD274, CD40LG, CFP, AOC3, CCL24, CCL26, AMBP, ANGPT1, ADAMTS13, CCL14, C1Q, NECTIN4, SERPINC1, MMP3, BDNF, AHSG, CCL22, CCL11, IFNA1, IL6R, IL7, MPO, PDGFA
IL1B, PCSK9, CFD, IL23A, ANGPT2, CXCL9, IL25, IL3, IL36B, MMP7, LGALS3BP, CCL2, IL12p40, IL2, ALDH1A1, IL1RL1, CCL23, GZMB, MMP2, ICAM1, REG3A, IL1A, KLK6, IL18, LGALS9, HGF, TSLP, KIT, CCL18, IL4, PTX3, IL5, IL8, TGFA, MMP13, SPP1, SFTPD, LBP, IL33, IL17A, IL15, MIA, MICA, BMP10, IFNG, IGFBP4, NPHS1, IFNL3, IL12p70, IL13, MUC16, MUC1, IL1RN, MADCAM1, IL11, IL10, LUM, LEP, LGALS3, LCN2, IL4R, MCAM, ESM1, IGFBP1, IGFBP2, MB, MBL2, IL6, S100A12, RETN, LIF
GDF15, FURIN, FSTL3, ENPP2, KITLG, EGF, FLT1, ERBB3, FLT3LG, FCER2, LRG1, F3, FGF2, FABP4, ERBB2, IL31, GZMA, GPNMB, FASLG, DPP4, GH1, FAP, MMP12, MIF
MG1
MG2

Figure S9. Heterogeneous inflammatory responses in moderate COVID-19 patients. **A**. Unsupervised hierarchical clustering based on all 191 proteins assayed in 745 plasma samples from 315 moderate COVID-19 patients showed clear separation of two groups, namely, MG1_S and MG2_S. The heatmap shows z scores arranged according to the days after symptom onset within the two groups after clustering. **B**. Unsupervised hierarchical clustering based on the mean concentration of 191 proteins assayed in 315 moderate COVID-19 patients showed clear separation of two groups, namely, MG1 and MG2.

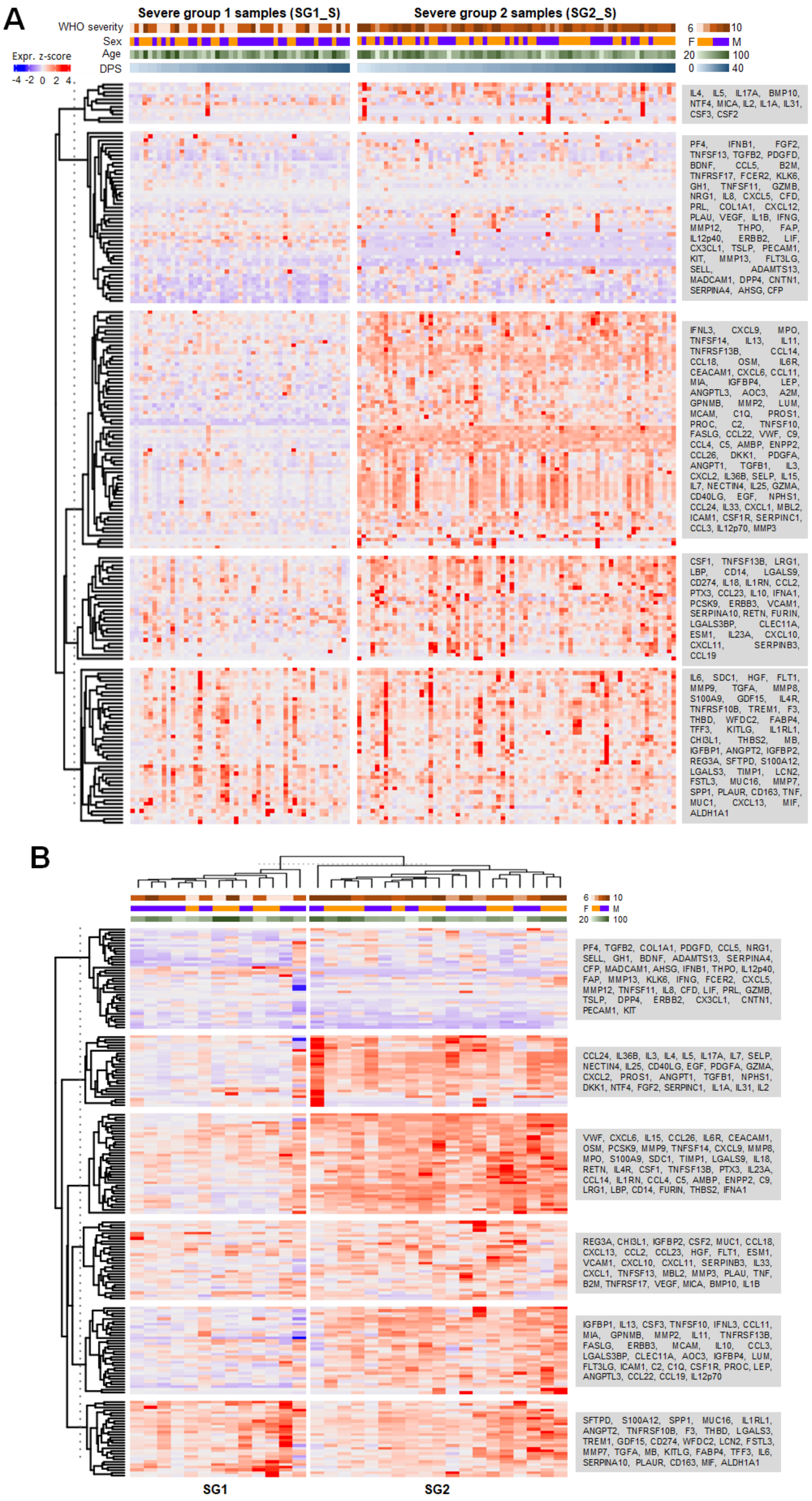
A
Severe group 1 samples (SG1_S)
Severe group 2 samples (SG2_S)
WHO severity
Sex
Age
DPS
Expr. z-score
-4 -2 0 2 4
6 10
F M
20 100
0 40
IL4, IL5, IL17A, BMP10, NTF4, MICA, IL2, IL1A, IL31, CSF3, CSF2
PF4, IFNB1, FGF2, TNFSF13, TGFB2, PDGFD, BDNF, CCL5, B2M, TNFRSF17, FCER2, KLK6, GH1, TNFSF11, GZMB, NRG1, IL8, CXCL5, CFD, PRL, COL1A1, CXCL12, PLAU, VEGF, IL1B, IFNG, MMP12, THPO, FAP, IL12p40, ERBB2, LIF, CX3CL1, TSLP, PECAM1, KIT, MMP13, FLT3LG, SELL, ADAMTS13, MADCAM1, DPP4, CNTN1, SERPINA4, AHSG, CFP
IFNL3, CXCL9, MPO, TNFSF14, IL13, IL11, TNFRSF13B, CCL14, CCL18, OSM, IL6R, CEACAM1, CXCL6, CCL11, MIA, IGFBP4, LEP, ANGPTL3, AOC3, A2M, GPNMB, MMP2, LUM, MCAM, C1Q, PROS1, PROC, C2, TNFSF10, FASLG, CCL22, VWF, C9, CCL4, C5, AMBP, ENPP2, CCL26, DKK1, PDGFA, ANGPT1, TGFB1, IL3, CXCL2, IL36B, SELP, IL15, IL7, NECTIN4, IL25, GZMA, CD40LG, EGF, NPHS1, CCL24, IL33, CXCL1, MBL2, ICAM1, CSF1R, SERPINC1, CCL3, IL12p70, MMP3
CSF1, TNFSF13B, LRG1, LBP, CD14, LGALS9, CD274, IL18, IL1RN, CCL2, PTX3, CCL23, IL10, IFNA1, PCSK9, ERBB3, VCAM1, SERPINA10, RETN, FURIN, LGALS3BP, CLEC11A, ESM1, IL23A, CXCL10, CXCL11, SERPINB3, CCL19
IL6, SDC1, HGF, FLT1, MMP9, TGFA, MMP8, S100A9, GDF15, IL4R, TNFRSF10B, TREM1, F3, THBD, WFDC2, FABP4, TFF3, KITLG, IL1RL1, CHI3L1, THBS2, MB, IGFBP1, ANGPT2, IGFBP2, REG3A, SFTPD, S100A12, LGALS3, TIMP1, LCN2, FSTL3, MUC16, MMP7, SPP1, PLAUR, CD163, TNF, MUC1, CXCL13, MIF, ALDH1A1
B
6 10
F M
20 100
PF4, TGFB2, COL1A1, PDGFD, CCL5, NRG1, SELL, GH1, BDNF, ADAMTS13, SERPINA4, CFP, MADCAM1, AHSG, IFNB1, THPO, IL12p40, FAP, MMP13, KLK6, IFNG, FCER2, CXCL5, MMP12, TNFSF11, IL8, CFD, LIF, PRL, GZMB, TSLP, DPP4, ERBB2, CX3CL1, CNTN1, PECAM1, KIT
CCL24, IL36B, IL3, IL4, IL5, IL17A, IL7, SELP, NECTIN4, IL25, CD40LG, EGF, PDGFA, GZMA, CXCL2, PROS1, ANGPT1, TGFB1, NPHS1, DKK1, NTF4, FGF2, SERPINC1, IL1A, IL31, IL2
VWF, CXCL6, IL15, CCL26, IL6R, CEACAM1, OSM, PCSK9, MMP9, TNFSF14, CXCL9, MMP8, MPO, S100A9, SDC1, TIMP1, LGALS9, IL18, RETN, IL4R, CSF1, TNFSF13B, PTX3, IL23A, CCL14, IL1RN, CCL4, C5, AMBP, ENPP2, C9, LRG1, LBP, CD14, FURIN, THBS2, IFNA1
REG3A, CHI3L1, IGFBP2, CSF2, MUC1, CCL18, CXCL13, CCL2, CCL23, HGF, FLT1, ESM1, VCAM1, CXCL10, CXCL11, SERPINB3, IL33, CXCL1, TNFSF13, MBL2, MMP3, PLAU, TNF, B2M, TNFRSF17, VEGF, MICA, BMP10, IL1B
IGFBP1, IL13, CSF3, TNFSF10, IFNL3, CCL11, MIA, GPNMB, MMP2, IL11, TNFRSF13B, FASLG, ERBB3, MCAM, IL10, CCL3, LGALS3BP, CLEC11A, AOC3, IGFBP4, LUM, FLT3LG, ICAM1, C2, C1Q, CSF1R, PROC, LEP, ANGPTL3, CCL22, CCL19, IL12p70
SFTPD, S100A12, SPP1, MUC16, IL1RL1, ANGPT2, TNFRSF10B, F3, THBD, LGALS3, TREM1, GDF15, CD274, WFDC2, LCN2, FSTL3, MMP7, TGFA, MB, KITLG, FABP4, TFF3, IL6, SERPINA10, PLAUR, CD163, MIF, ALDH1A1
SG1
SG2

Figure S10. Heterogeneous inflammatory responses in severe COVID-19 patients. **A**. Unsupervised hierarchical clustering based on all 191 proteins assayed in 130 plasma samples from 32 severe COVID-19 patients showed clear separation of two groups, namely, SG1_S and SG2_S. The heatmap shows z scores arranged according to the days after symptom onset within the two groups after clustering. **B**. Unsupervised hierarchical clustering based on the mean concentration of 191 proteins assayed in 32 severe COVID-19 patients showed clear separation of two groups, namely, SG1 and SG2.

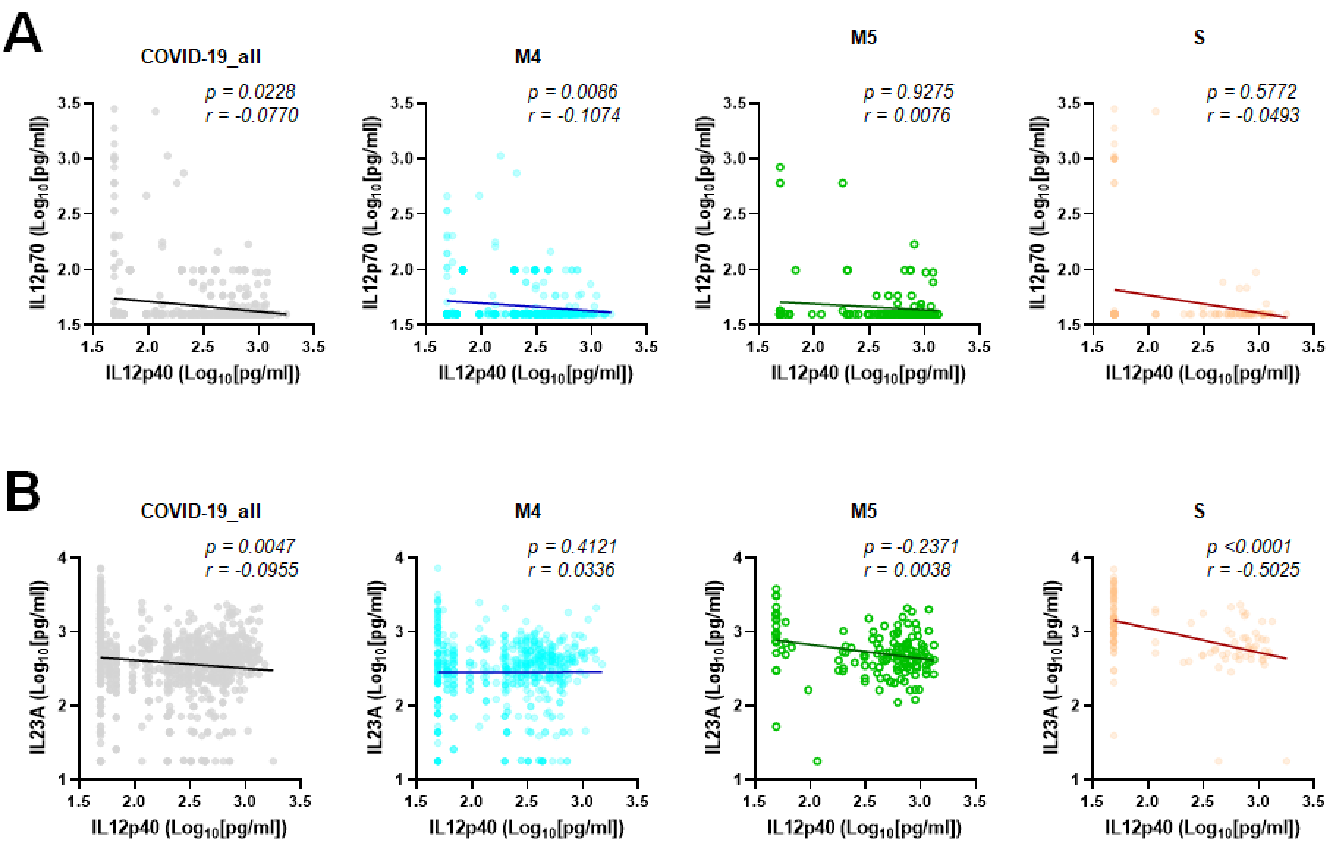

Figure S11. Correlation of IL12p40 with IL12p70 and IL23 in COVID-19 patients. A. Correlation plots of IL12p40 with IL12p70 in COVID-19 patients (COVID-19_all) and COVID-19 patients with various disease severities (M4 and M5: moderate pneumonia, S: severe pneumonia). **B.** Correlation plots of IL12p40 with IL23A in COVID-19 patients (COVID-19_all) and COVID-19 patients with various disease severities (M4 and M5: moderate pneumonia, S: severe pneumonia).
